# Distinct metabolomic signatures in allergic rhinitis with concurrent chronic spontaneous urticaria: an untargeted metabolomics analysis reveals novel biomarkers and pathway alterations

**DOI:** 10.3389/fimmu.2025.1555664

**Published:** 2025-06-10

**Authors:** Xiaohong Lyu, Yi Liu, Huaijun Zheng, Hongna Li, Zhoujie Wu, Yi Sun, Shanhong Wu, Xuehan Jiang, Shandong Wu, Rui Tang, Yue Gao, Jinlyu Sun

**Affiliations:** ^1^ Department of Allergy, Beijing Key Laboratory of Precision Medicine for Diagnosis and Treatment of Allergic Diseases, National Clinical Research Center for Dermatologic and Immunologic Diseases, Peking Union Medical College Hospital, Chinese Academy of Medical Sciences, Peking Union Medical College, Beijing, China; ^2^ Department of Breast Surgery, Peking Union Medical College Hospital, Chinese Academy of Medical Sciences, Peking Union Medical College, Beijing, China; ^3^ Hangzhou Zheda Dixun Biological Gene Engineering Co.,Ltd., Hangzhou, China; ^4^ Eight-year Clinical Medicine Program, Peking Union Medical College, Chinese Academy of Medical Sciences, Beijing, China; ^5^ Pulmonary and Critical Care Medicine, Binzhou People’s Hospital, Binzhou, China; ^6^ Zhejiang Key Laboratory of Traditional Chinese Medicine for the Prevention and Treatment of Senile Chronic Diseases, Affiliated Hangzhou First People’s Hospital, School of Medicine, Westlake University, Hangzhou, China

**Keywords:** allergic rhinitis, chronic spontaneous urticaria, metabolomics, biomarkers, pathway analysis

## Abstract

**Background:**

Although allergic rhinitis (AR) and chronic spontaneous urticaria (CSU) share overlapping immunological pathways, their distinct clinical manifestations imply the involvement of unique underlying mechanisms. This study aimed to investigate the metabolomic differences between patients with AR alone and those with concurrent AR and CSU (AR_CSU, defined as patients simultaneously presenting with both conditions).

**Methods:**

An untargeted metabolomic analysis was performed on serum samples from 53 AR patients and 14 AR_CSU patients using ultra-performance liquid chromatography coupled with high-resolution mass spectrometry (LC-MS/MS). Multivariate statistical analyses, including partial least squares-discriminant analysis (PLS-DA) and orthogonal partial least squares-discriminant analysis (OPLS-DA), were employed to identify differential metabolites and metabolic pathways.

**Results:**

A total of 209 significantly different metabolites were identified between the AR and AR_CSU groups (p < 0.05). Distinct metabolic patterns were observed through PLS-DA and OPLS-DA analyses, with no overlap between the two groups. Twenty metabolites exhibited high diagnostic potential (AUC > 0.75), among which Fasciculic acid C and Biotin-XX-Hydrazide showed particularly strong discriminatory power (AUC ≈ 0.8). Pathway analysis highlighted significant alterations in linoleic acid, fatty acid, and arachidonic acid metabolism. Notably, fatty acid elongation pathways were upregulated in AR_CSU patients, whereas primary bile acid biosynthesis and Fc gamma R-mediated phagocytosis were downregulated.

**Conclusions:**

This study represents the first comprehensive metabolomic comparison between AR and AR_CSU, identifying distinct metabolic signatures and potential biomarkers. These findings advance our understanding of the pathophysiological differences between these conditions and could inform the development of targeted therapeutic strategies.

## Introduction

Allergic diseases, including allergic rhinitis (AR) and chronic spontaneous urticaria (CSU), impose a substantial global health burden, with AR affecting approximately one-third of the global population and demonstrating a rising prevalence in China over the past decade (1, 2). Although precise global epidemiological data for CSU remain unavailable, existing studies estimate its prevalence at 0.5% to 1% of the population (3). Despite their prevalence, the development of accurate biomarkers and effective treatments has not kept pace with the growing burden of these conditions, leaving healthcare providers with suboptimal diagnostic tools and patients reliant on allergen avoidance or antihistamine medications for symptom management.

Allergic disorders are multifaceted conditions arising from the interplay of genetic, epigenetic, and environmental factors (4). Allergic diseases frequently present as the sequential development of one allergic condition following another, such as the onset of chronic spontaneous urticaria (CSU) in individuals with pre-existing allergic rhinitis (AR). When both conditions occur simultaneously in the same patient (referred to as AR_CSU in this study), they present unique diagnostic and therapeutic challenges. While both AR and CSU are characterized by type 2 immune response mechanisms (5), their distinctly diverse clinical manifestations indicate the involvement of additional unique pathogenic processes contributing to their heterogeneous phenotypes. A deeper understanding of these pathogenic mechanisms could facilitate the development of urgently needed biomarkers and novel therapeutic strategies.

While previous studies have investigated these conditions separately, there is a pressing need to understand the distinct metabolic signatures that differentiate AR from AR_CSU. Metabolomics offers a powerful approach to address these knowledge gaps for several reasons. First, as a comprehensive analysis of small molecules in biological systems, it can reveal alterations in metabolic pathways that may not be apparent through traditional immunological analyses. Second, metabolomic profiling can detect changes driven by both genetic and environmental factors, making it particularly valuable for complex allergic disorders where multiple factors influence disease manifestation. Finally, metabolomics has already demonstrated success in identifying novel biomarkers in other allergic conditions, suggesting its potential utility in distinguishing AR from AR_CSU. While previous metabolomic studies have explored metabolic alterations in AR, few have specifically investigated CSU or the combined condition of AR_CSU.

Untargeted metabolomic profiling has emerged as a robust approach for elucidating altered pathways underlying complex diseases, including AR and CSU (6, 7). Its capacity to detect alterations driven by genetic and environmental factors renders it particularly valuable for uncovering disease mechanisms and identifying biomarkers in these complex atopic disorders. Several studies have utilized untargeted metabolomics to investigate AR, identifying metabolic signatures linked to its pathogenesis and severity. For example, a recent study identified significant alterations in amino acid, lipid, and energy metabolism in AR patients, highlighting potential therapeutic targets (8). In contrast, fewer studies have explored untargeted metabolomics in CSU, particularly in the context of AR_CSU. Notably, one study demonstrated alterations in gut microbiome composition and metabolome in CSU patients, suggesting a potential role for gut dysbiosis in CSU pathogenesis (9). More recently, a comprehensive analysis combining microbiome and metabolome data revealed distinct gut microbial and metabolic profiles in CSU patients, potentially contributing to disease development and progression (10). These findings underscore the potential of metabolomics in identifying disease-specific signatures and novel therapeutic targets for allergic conditions.

In this pilot study, we performed untargeted metabolomic profiling in patients with AR and AR_CSU to better define the metabolic changes associated with each clinical entity. The aim of this study was to discover possible metabolite alterations and possible metabolic pathway alterations in CSU in combination with AR. Our study uniquely addresses this gap by identifying distinct metabolic signatures in AR_CSU, which have not been previously characterized. This study investigates the unique metabolic alterations in patients with concurrent AR and CSU, aiming to identify distinct endotypes and potential biomarkers that arise from their coexistence, rather than implying a direct causal link or shared immune mechanism.

## Methods

### Study population

This study included 67 participants, comprising 53 patients with AR alone and 14 patients with concurrent AR and CSU (AR_CSU). Participants were recruited from the Peking Union Medical College Hospital cohort. The study was approved by the Peking Union Medical College Hospital IRB (Number: K3949).

The diagnosis of AR was established by the treating physician based on a combination of positive skin prick tests or specific IgE tests, along with a history of two or more nasal symptoms (rhinorrhea, nasal obstruction, sneezing, or nasal pruritus) persisting for more than one hour on most days (11). CSU was diagnosed based on international guidelines, characterized by recurrent wheals, angioedema, or both lasting for more than six weeks without any identifiable external trigger (3). Patients with AR_CSU were defined as individuals meeting the diagnostic criteria for both AR and CSU. AR severity was assessed using the Total Nasal Symptom Score (TNSS), whereas CSU severity was evaluated using the Urticaria Activity Score over 7 days (UAS7) (12, 13). All CSU patients included had active disease at the time of sample collection, defined as a UAS7 score ≥16. Patients had not received systemic corticosteroids or immunosuppressive therapies within the previous 4 weeks.

Patients requiring systemic corticosteroids were excluded. Non-atopic controls were defined as individuals with no personal history of allergic conditions. Patients with autoimmune diseases were carefully evaluated rather than broadly excluded, ensuring that only conditions known to significantly impact metabolomic profiles were excluded. Additionally, patients who received allergen-specific immunotherapy within the past year (instead of five years) were excluded to balance the need for sample size and control potential confounding effects (11).

### Sample preparation and extraction

#### Liquid samples class I

Patients were instructed to fast before blood sample collection to minimize dietary influences on serum metabolites. Comorbidities, medication use, and dietary influences were controlled by including only patients with AR and CSU, excluding those with other conditions, and ensuring fasting before blood collection.

Blood samples were collected in serum separator tubes following the recommended handling guidelines for metabolomics studies and stored at -80°C within 2 hours of collection. The serum samples were thawed on ice and vortexed for 10 seconds. A 50 μL aliquot of serum was mixed with 300 μL of extraction solution (ACN: Methanol = 1:4, v/v) containing internal standards in a 2 mL microcentrifuge tube. The mixture was vortexed for 3 minutes and centrifuged at 12,000 rpm for 10 minutes at 4°C. A 200 μL aliquot of the supernatant was collected, incubated at -20°C for 30 minutes, and centrifuged at 12,000 rpm for 3 minutes at 4°C. Finally, a 180 μL aliquot of the supernatant was transferred for LC-MS analysis.

### Metabolomic profiling

Global, non-targeted metabolomic profiling was conducted using ultra-performance liquid chromatography (LC-30A, Shimadzu, Japan) coupled with a high-resolution/accurate mass spectrometer (TripleTOF 6600+, SCIEX, Foster City, CA, USA) in LC-MS/MS mode. Two separate LC/MS methods were applied to all samples. One aliquot was analyzed under positive ionization conditions and eluted using a T3 column (Waters ACQUITY Premier HSS T3 Column, 1.8 µm, 2.1 mm × 100 mm) with a mobile phase consisting of 0.1% formic acid in water (solvent A) and 0.1% formic acid in acetonitrile (solvent B). The gradient program was as follows: 5% to 20% solvent B over 2 minutes, increased to 60% over the next 3 minutes, further increased to 99% within 1 minute and held for 1.5 minutes, followed by a return to 5% solvent B within 0.1 minutes and held for 2.4 minutes. The analytical conditions included a column temperature of 40°C, a flow rate of 0.4 mL/min, and an injection volume of 4 μL. A second aliquot was analyzed under negative ionization conditions using the same elution gradient as in the positive mode.

### MS conditions (AB)

Data acquisition was performed in information-dependent acquisition (IDA) mode using Analyst TF 1.7.1 Software (Sciex, Concord, ON, Canada). The source parameters were configured as follows: ion source gas 1 (GAS1), 50 psi; ion source gas 2 (GAS2), 50 psi; curtain gas (CUR), 25 psi; source temperature (TEM), 550°C; declustering potential (DP), 60 V for positive mode and −60 V for negative mode; and ion spray voltage floating (ISVF), 5000 V for positive mode and −4000 V for negative mode. The TOF MS scan parameters were set to a mass range of 50–1000 Da, with an accumulation time of 200 ms and dynamic background subtraction enabled. For product ion scans, the parameters were as follows: mass range, 25–1000 Da; accumulation time, 40 ms; collision energy, 30 V for positive mode and −30 V for negative mode; collision energy spread, 15; resolution, UNIT; charge state, 1 to 1; intensity threshold, 100 cps; exclusion of isotopes within 4 Da; mass tolerance, 50 ppm; and a maximum of 18 candidate ions monitored per cycle.

### Statistical analysis

The raw data files acquired from LC-MS were converted into mzXML format using ProteoWizard software. Peak extraction, alignment, and retention time correction were performed using the XCMS program. The “SVR” method was applied to correct the peak areas. Peaks with a detection rate lower than 50% in each sample group were excluded. Metabolite identification was subsequently conducted by querying the laboratory’s self-built database, integrated public databases, AI databases, and metDNA. Unsupervised principal component analysis (PCA) was performed using the prcomp function in R (www.r-project.org), with unit variance scaling applied to the data beforehand. Hierarchical cluster analysis (HCA) results for samples and metabolites were visualized as heatmaps with dendrograms, while Pearson correlation coefficients (PCCs) between samples were calculated using the cor function in R and presented as heatmaps. Both HCA and PCC analyses were carried out using the R package ComplexHeatmap. For HCA, normalized signal intensities of metabolites (scaled to unit variance) were visualized as a color gradient.

For two-group analysis, differential metabolites were determined by VIP (VIP > 1) and P-value (P-value < 0.05, Student’s t test). A two-sided Student’s t-test was used for statistical analysis. VIP values were extracted from OPLS-DA result, which also contain score plots and permutation plots, was generated using R package MetaboAnalystR. The data was log transform (log2) and mean centering before OPLS-DA. In order to avoid overfitting, a permutation test (200 permutations) was performed. Identified metabolites were annotated using KEGG Compound database (http://www.kegg.jp/kegg/compound/), annotated metabolites were then mapped to KEGG Pathway database (http://www.kegg.jp/kegg/pathway.html). Significantly enriched pathways are identified with a hypergeometric test’s P-value for a given list of metabolites. R (base package, version 4.1.2), R (corrplot, version 0.92), R (ComplexHeatmap, version 2.9.4), R (MetaboAnalystR, version 1.0.1), R (fmsb, version 0.7.1), R (igraph; ggraph, version 1.2.11; 2.0.5), R (igraph, version 1.2.11) and R (FELLA, version 1.2.0) were used in this study.

## Results

A total of 67 patients were enrolled in this study, including 53 patients diagnosed with allergic rhinitis (AR) and 14 patients with chronic spontaneous urticaria combined with AR (AR_CSU). The baseline characteristics of the two groups are summarized in **Table 1**. The mean age of AR patients (32.82 ± 8.88 years) was significantly lower than that of AR_CSU patients (38.54 ± 9.23 years) (*P* = 0.044). A correlation analysis was conducted to assess the influence of age on metabolomic profiles, showing no significant correlation. No significant differences were observed in gender distribution between the groups, with males comprising 26.0% of AR patients and 30.8% of AR_CSU patients (*P* = 0.730). Similarly, there were no significant differences in rhinitis scores, maximum medical history, seasonal allergy prevalence, allergy to specific allergens, family history of allergies, or the effectiveness of antihistamine medication between the two groups.

**Table 1:** Summary characteristics of the study population.

Characteristics	AR (n=53)	AR_CSU (n=14)	P-value*
Age, year	32.82 ± 8.88	38.54 ± 9.23	**0.044**
Sex, male, n, %	13 (26.0%)	4 (30.8%)	0.730
Rhinitis Score	2.78 ± 0.47	2.92 ± 0.29	0.324
Maximum medical history, years	7.07 ± 6.04	10.00 ± 6.62	0.132
Seasonal allergies, n, %	10 (20.4%)	2 (15.4%)	0.684
Allergy to specific allergens, n, %	13 (24.5%)	5 (35.7%)	0.566
Family history of allergies, n, %	18 (35.3%)	8 (61.5%)	0.085
Antihistamine medication effective, n, %	44 (83.0%)	13 (92.9%)	0.634

* and Bold value suggests p<0.05 for statistically different results.

A total of 3,998 biochemicals were analyzed in this study. The metabolites corresponding to the codes can be found in **Supplementary File 1**. Comparative analysis between AR and AR_CSU patients identified 209 metabolites with significant differences, based on a cutoff *P* value of <0.05. In this study, untargeted metabolomics profiling using LC-MS was conducted to explore the differences in metabolomic patterns between AR and AR_CSU patients. Metabolomic data from the two groups were analyzed using multivariate statistical approaches, including Partial Least Squares Discriminant Analysis (PLS-DA) and Orthogonal Partial Least Squares Discriminant Analysis (OPLS-DA). These multivariate methods facilitated the extraction of meaningful information from the complex metabolomic datasets, enabling dimensionality reduction and clear visual differentiation between AR and AR_CSU patients.

**Figure 1:**
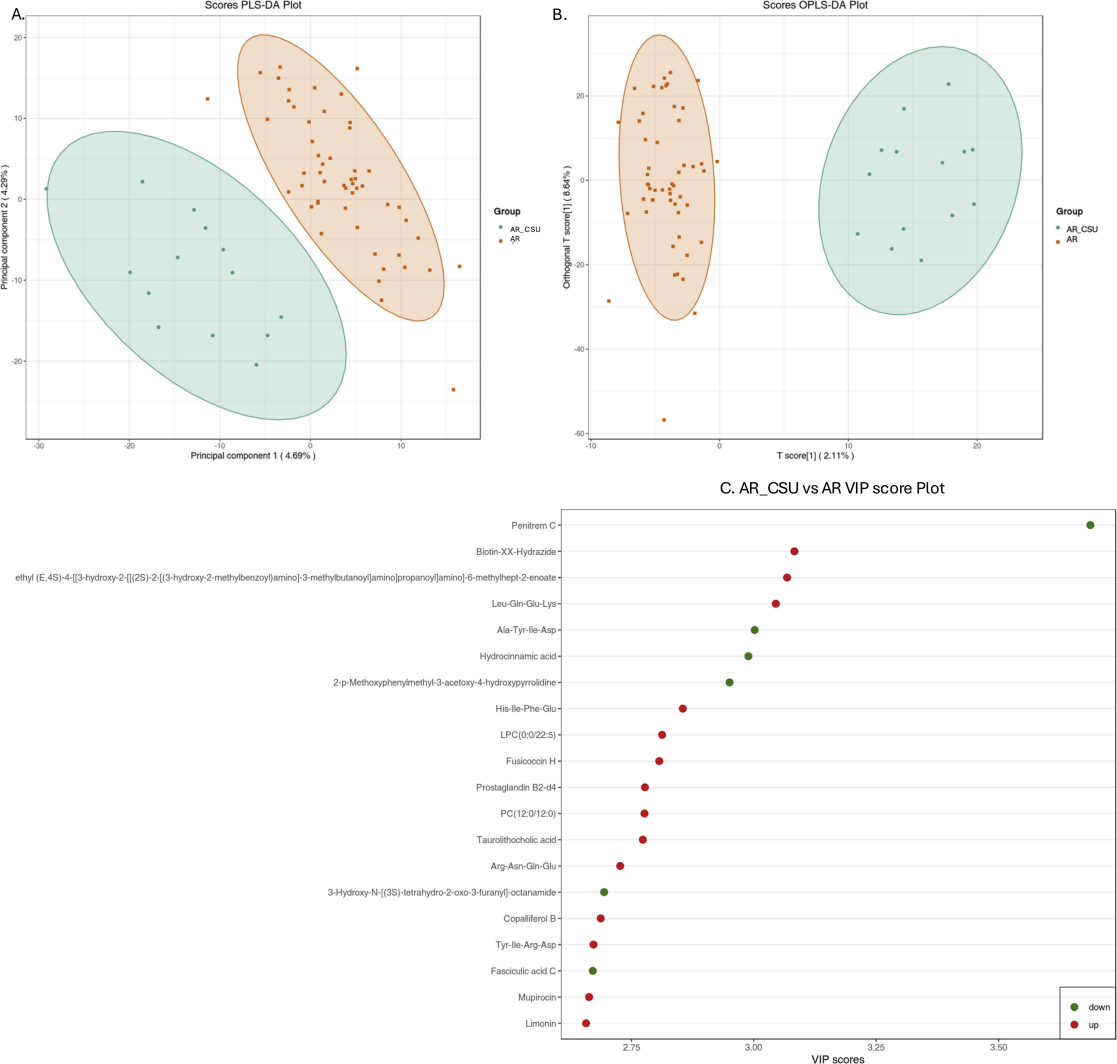
Multivariate statistical analysis of metabolic data from AR and AR_CSU patients. **(A)** The score plot generated by Partial Least Squares Discriminant Analysis (PLS-DA) and **(B)** the score plot produced by Orthogonal Partial Least Squares Discriminant Analysis (OPLS-DA) clearly illustrate distinct metabolomic profiles between AR and AR_CSU patients. **(C)** The VIP score plot, derived from OPLS-DA, highlights the top 20 metabolites that show significant differences between AR and AR_CSU patients. The colored boxes on the right side of the plot represent the relative concentration variations of the corresponding metabolites, with green indicating low concentrations and red representing high concentrations.

As illustrated in **Figure 1**, a clear separation between the two groups was observed in both the PLS-DA (**Figure 1A**) and OPLS-DA (**Figure 1B**) models. In the PLS-DA plot, all AR samples (red dots) were clustered on the right, while all AR_CSU samples (green dots) were grouped on the left. Similarly, the OPLS-DA plot showed the two sets of dots in opposite positions, with no overlap observed (**Figures 1A, B**). These results indicate significant differences in the metabolomic profiles between AR and AR_CSU patients.

The VIP plot, derived from the OPLS-DA model, highlights the top 20 compounds with VIP scores greater than 1, ranked according to their discriminatory power (**Figure 1C**). Variables with VIP scores exceeding 1 are considered highly influential. The principal compounds identified as most critical for group segregation include Penitrem C, Biotin-XX-Hydrazide, ethyl (E,4S)-4-((3-hydroxy-2-(((2S)-2-((3-hydroxy-2-methylbenzoyl)amino)-3-methylbutanoyl)amino)propanoyl)amino)-6-methylhept-2-enoate, Leu-Gln-Glu-Lys, Ala-Tyr-Ile-Asp, Hydrocinnamic acid, 2-p-Methoxyphenylmethyl-3-acetoxy-4-hydroxypyrrolidine, His-Ile-Phe-Glu, LPC(0:0/22:5), Fusicoccin H, Prostaglandin B2-d4, PC(12:0/12:0), Taurolithocholic acid, Arg-Asn-Gln-Glu, 3-Hydroxy-N-((3S)-tetrahydro-2-oxo-3-furanyl)-octanamide, Copalliferol B, Tyr-Ile-Arg-Asp, Fasciculic acid C, Mupirocin, and Limonin.

**Figure 2:**
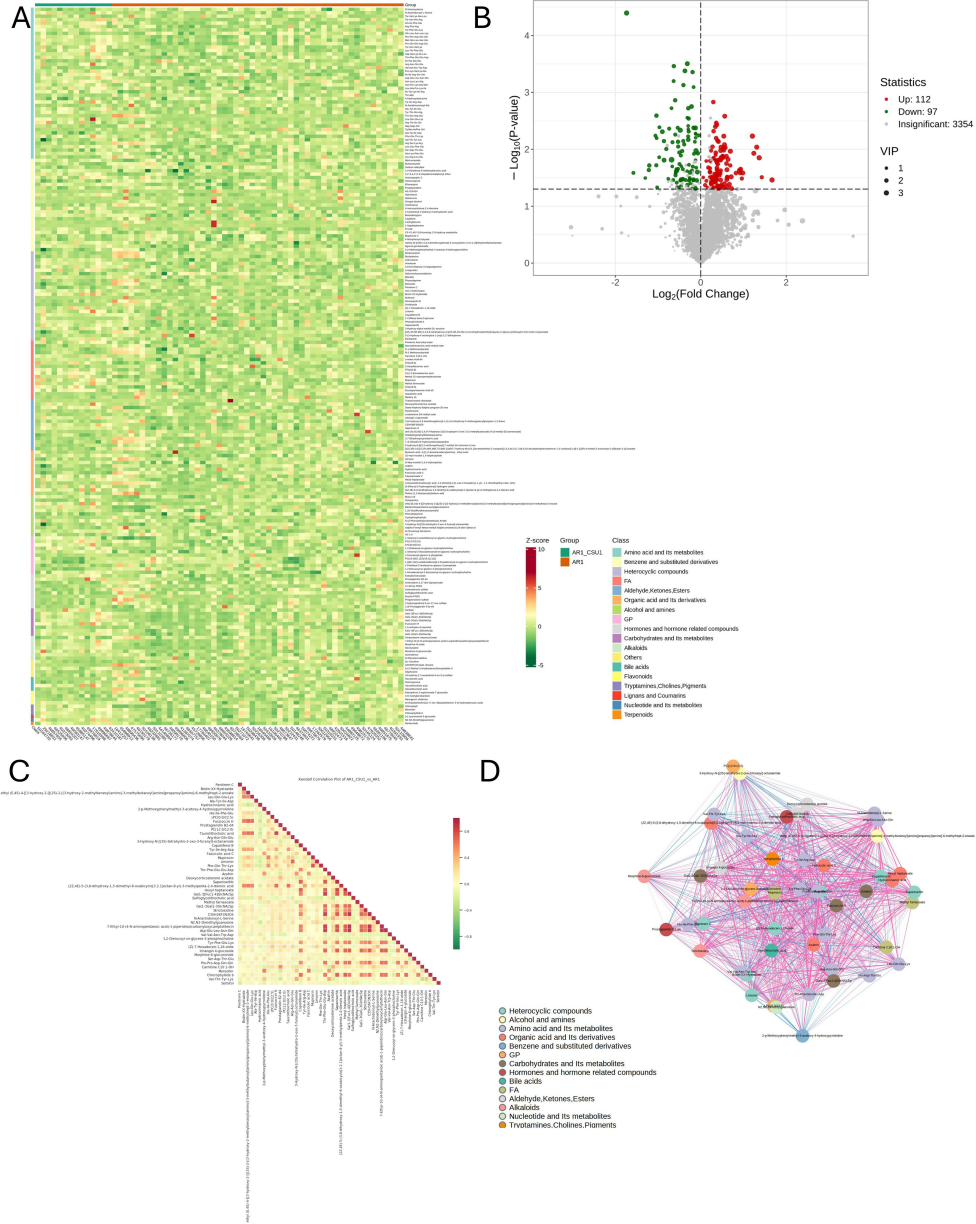
Variations in metabolite levels between AR and AR_CSU patients. **(A)** The heatmap displays the relative abundances of the top 50 metabolites and lipids between AR and AR_CSU patients. **(B)** The volcano plot identifies metabolites with significant differences between the two groups based on p-values. **(C)** The differential metabolite correlation heatmap illustrates the Pearson correlation coefficients between metabolites. **(D)** The differential metabolite correlation network diagram visualizes the relationships among significantly different metabolites.

**Figure 2** illustrates variations in metabolite levels between AR and AR_CSU patients. Specifically, a heatmap (**Figure 2A**) visualizes the relative abundances of the top 50 significant metabolites, with color intensity representing concentration differences between AR and AR_CSU patients. A volcano plot (**Figure 2B**) highlights metabolites with significant differences between the two groups, where green, red, and gray points indicate downregulated, upregulated, and non-significant metabolites, respectively. There were 112 up metabolites and 97 down metabolites in AR_CSU group compared to AR group. The differential metabolite correlation heatmap (**Figure 2C**) and correlation network diagram (**Figure 2D**) further reveal the relationships and connectivity among significant metabolites, with color gradients and line thickness indicating the strength and nature of correlations.

**Figure 3:**
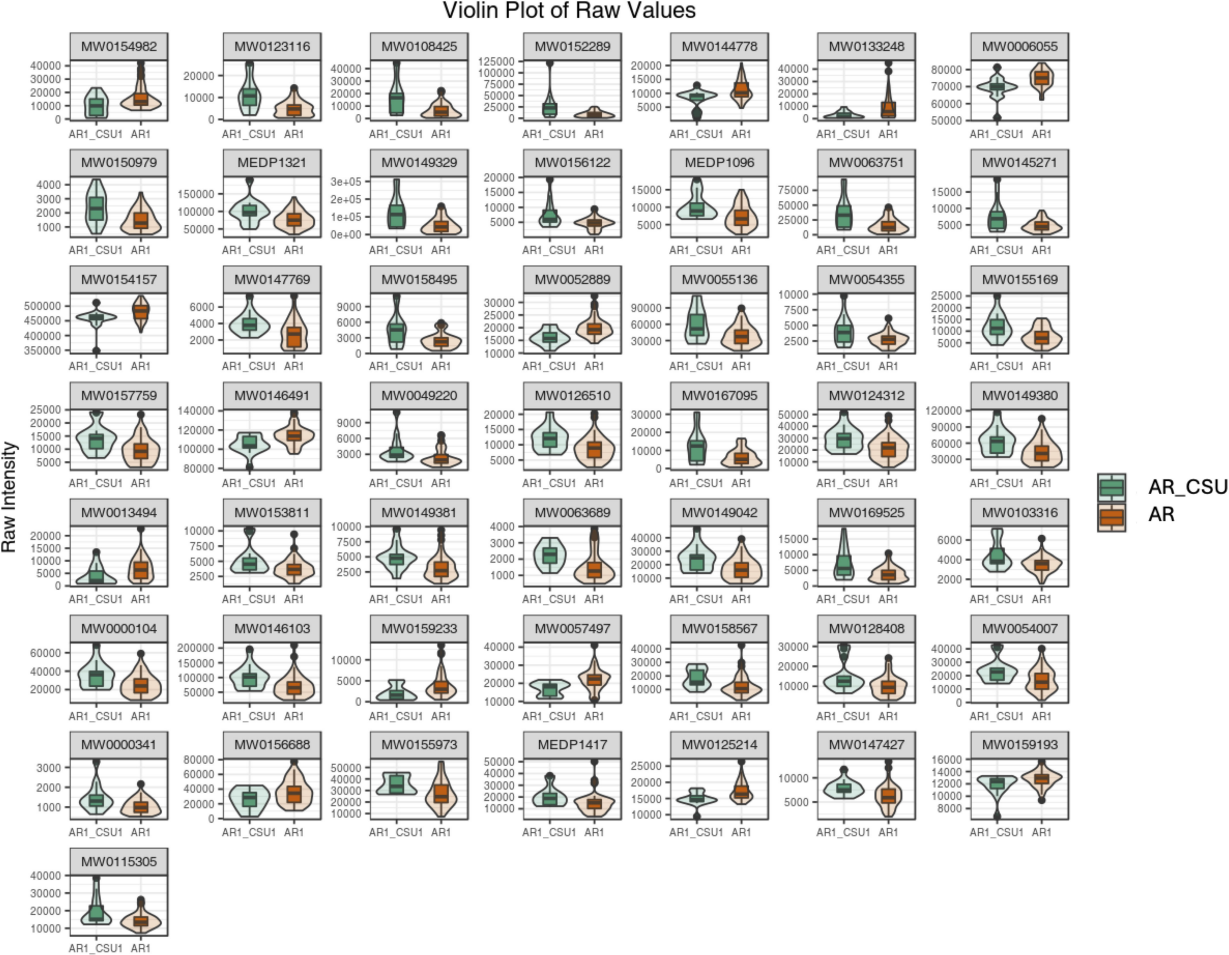
A violin plot combines features of a boxplot and a density plot, providing a comprehensive visualization of data distributions and their probability densities. The central box represents the interquartile range (IQR), the thin black line extending from the box denotes the 95% confidence interval, and the black horizontal line within the box indicates the median. The outer shape illustrates the density of the data distribution. The x-axis represents sample groupings, while the y-axis denotes the relative abundance of differential metabolites (raw peak area). P-values were calculated using the Student’s t-test.

Subsequently, the top 50 statistically significant metabolites were identified by combining results from the VIP plot (VIP score > 1) and volcano plot analysis (p-value < 0.05, Student’s t-test), as shown in **Figure 3**; **Supplementary File 2**. Specifically, 13 compounds exhibited decreased concentrations, while 37 compounds showed increased concentrations in AR_CSU compared to AR patients. A violin plot analysis was performed to visualize the distribution of peak intensities for these metabolites, combining box-and-line plots with density plots to illustrate data distributions and probability densities. Statistical significance was determined using Student’s t-test.

**Figure 4:**
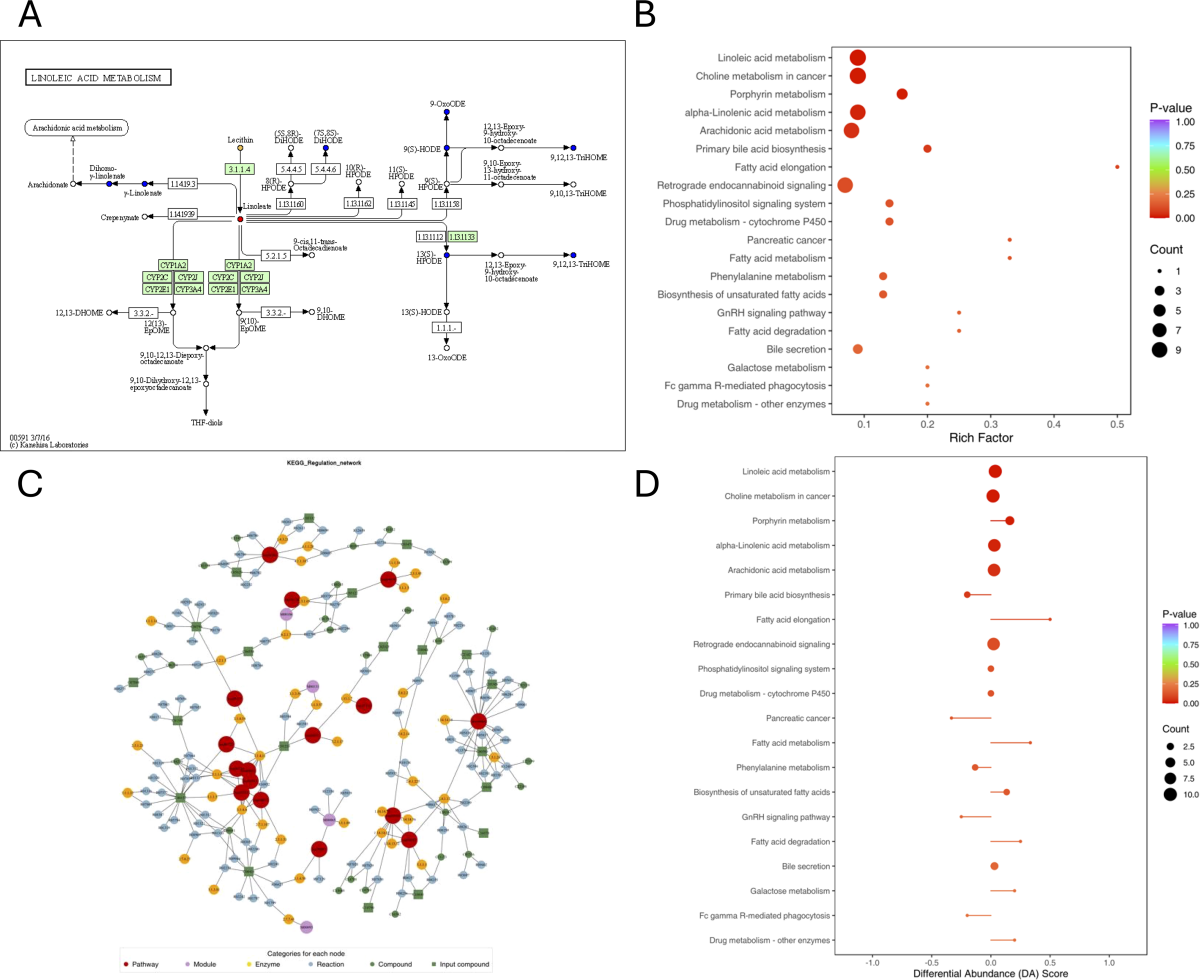
Pathways associated with each identified compound, as determined using the Kyoto Encyclopedia of Genes and Genomes (KEGG) pathway database. **(A)** KEGG pathway map of differential metabolites: Red indicates metabolites significantly upregulated in the experimental group, blue indicates metabolites detected without significant changes, green indicates metabolites significantly downregulated in the experimental group, and orange indicates pathways containing both upregulated and downregulated metabolites. **(B)** KEGG enrichment analysis of differential metabolites. The top 20 pathways were ranked by p-value and displayed in ascending order. **(C)** Network plot based on the KEGG database for the corresponding species. **(D)** Differential Abundance (DA) score plot. A score of 1 indicates that all identified metabolites in the pathway are upregulated, while a score of -1 indicates that all identified metabolites are downregulated.

**Figure 4** also lists the corresponding pathways that each identified compound was involved in, based on the knowledge derived from Kyoto Encyclopedia of Genes and Genomes (KEGG) pathway database. The represent differentially expressed compounds found between AR and AR_CSU patients belonged to **Figure 4A** (linoleic acid metabolism, other pathways could be found in **Supplementary Files 4**). Metabolic pathways were used to find the causes of phenotypic differences among the study subjects. Differential metabolite KEGG enrichment analysis was shown in **Figure 4B**. The Pathways included linoleic acid metabolism, choline metabolism in cancer, porphyrin metabolism, alpha-linolenic acid metabolism, arachidonic acid metabolism, primary bile acid biosynthesis, fatty acid elongation, retrograde endocannabinoid signaling, drug metabolism- cytochrome p450, pancreatic cancer, fatty acid metabolism, phenylalanine metabolism, biosynthesis of unsaturated fatty acids, GnRH signaling pathway, fatty acid degradation, bile secretion, galactose metabolism, fc gamma r-mediated phagocytosis and drug metabolism - other enzymes. After obtaining the matching information of the differential metabolites, the pathway search and regulatory interactions network analysis were performed based on the KEGG database of the corresponding species (**Figure 4C**), which is presented in a network plot (network plot). **Figure 4D** suggests that the Fatty acid elongation metabolic pathway is up-regulated in the AR_CSU group as a whole, in addition to fatty acid metabolism, fatty acid degradation, galactose metabolism and drug metabolism-other enzymes.

**Figure 5:**
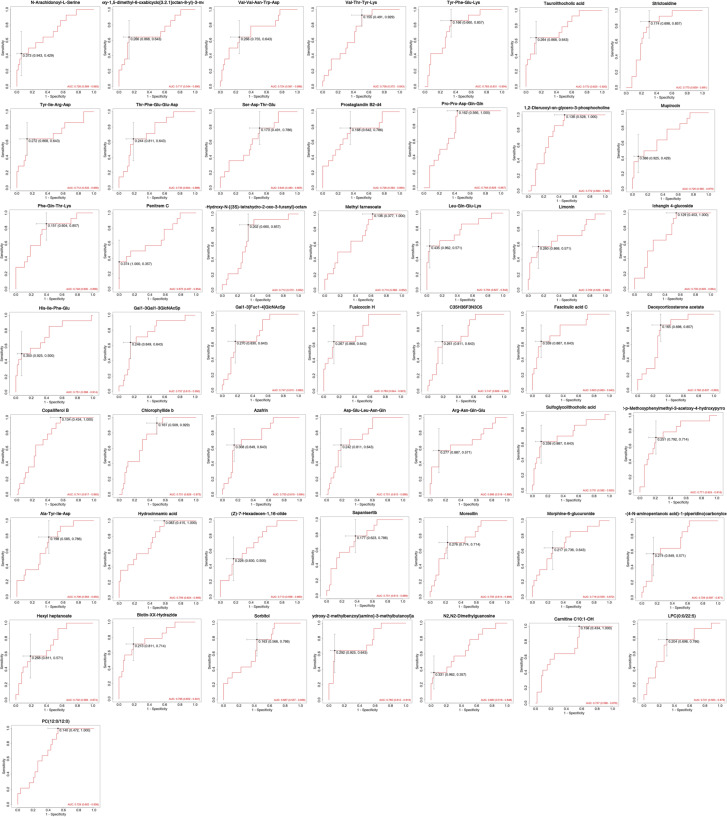
ROC curve analysis of statistically significant compounds.

The differentially expressed metabolites were examined by ROC curves, aimed at evaluating the diagnostic performances of potential biomarkers, including sensitivity and specificity (**Figure 5**). The area under the ROC curve (AUC) was used to detect the accuracy and efficiency of this method in distinguishing AR and AR_CSU patients. Our data revealed that, among all the differentially expressed compounds, 20 exhibited the highest AUC (AUC>0.75). In particular, Fasciculic acid C and Biotin-XX-Hydrazide had AUC values around 0.8, indicating a good discriminating capability between AR and AR_CSU patients (**Figure 5**). Other compounds, including Leu-Gln-Glu-Lys, Fusicoccin H, Strictosidine, Taurolithocholic acid, 1,2-Dierucoyl-sn-glycero-3-phosphocholine, 2-p-Methoxyphenylmethyl -3-acetoxy-4-hydroxypyrrolidine, ethyl (E,4S)-4-((<3-hydroxy-2- (((2S)-2- ((3-hydroxy-2-methylbenzoyl)amino) -3, methylbutanoyl) amino) propanoyl) amino) -6-methylhept-2-enoate, Tyr-Phe-Glu-Lys, Deoxycorticosterone acetate, Hydrocinnamic acid, Gal1-3Gal1-3GlcNAcSp, Azafrin, Moreollin, His-Ile-Phe-Glu, Sapanisertib, Sulfoglycolithocholic acid, Asp-Glu-Leu-Asn-Gln, and Chlorophyllide b exhibited AUC values around or greater than 0.75, showing moderate abilities in the differentiation between the two group (**Figure 5**).

## Discussion

This study represents a comprehensive metabolomic investigation aimed at elucidating the distinct metabolic profiles of patients with allergic rhinitis (AR) and those with concurrent AR and chronic spontaneous urticaria (AR_CSU). To the best of our knowledge, this is the first study to systematically explore the metabolic differences between these two conditions (3, 11, 14), offering novel insights into their underlying pathophysiological mechanisms and potential biomarkers.

Multivariate statistical analyses, including PLS-DA and OPLS-DA, demonstrated clear and significant metabolic distinctions between AR and AR_CSU patients. The well-defined separation between groups, with no observed overlap, suggests fundamentally distinct metabolic signatures underlying these conditions. This finding is particularly significant, as it suggests that the presence of CSU in AR patients is not merely an additional symptom but instead represents a distinct pathophysiological state characterized by unique metabolic features.

This study identified potential biomarkers with high diagnostic value. Previous metabolomic research in allergic diseases has primarily focused on isolated conditions, often lacking comprehensive pathway analysis. Our study advances this field by integrating untargeted metabolomics with pathway analysis, revealing novel insights into lipid metabolism pathways specifically altered in CSU, such as arachidonic acid and linoleic acid metabolism. These findings underscore the complex pathophysiology of CSU and its interaction with AR, offering potential biomarkers for improved diagnosis and treatment.

Fasciculic acid C and Biotin-XX-Hydrazide exhibited strong discriminatory capabilities, with AUC values approaching 0.8, underscoring their potential utility as diagnostic markers. These findings hold significant clinical implications, potentially facilitating more accurate diagnoses and personalized treatment strategies. Fasciculic acid C has not previously been linked to allergic conditions. Its elevated levels in one of these conditions (AR or CSU) may reflect a novel aspect of immune responses or microbial interactions in allergic disorders (15). Biotin-XX-Hydrazide, a biotin derivative, may indicate altered biotin metabolism or signaling pathways in these allergic conditions. Biotin is known to play a critical role in immune function and inflammation (16), suggesting that alterations in biotin-related metabolites may reflect distinct immunomodulatory processes differentiating AR_CSU from AR.

Several metabolic pathways were found to be differentially regulated between AR and AR_CSU, shedding light on the distinct inflammatory and immunological mechanisms underlying these conditions, including Lipid related metabolism. Our findings revealed significant alterations in lipid metabolism pathways, particularly in linoleic acid and arachidonic acid metabolism. These changes may have profound implications for disease progression and symptom severity. Recent studies have shown that disruptions in lipid metabolism can affect multiple aspects of allergic inflammation, including barrier function, inflammatory mediator production, mast cell activation. Altered lipid metabolism can compromise epithelial barrier function, potentially exacerbating both AR and CSU symptoms. Studies by Miyata et al. (17) have demonstrated that specialized pro-resolving mediators derived from omega-3 fatty acids play crucial roles in maintaining epithelial integrity and resolving allergic inflammation. The upregulation of fatty acid elongation pathways in AR_CSU patients may lead to increased production of pro-inflammatory lipid mediators. This is supported by work from Dennis et al. (18), who showed that dysregulation of eicosanoid metabolism contributes to chronic inflammation in allergic diseases. Lipid mediators, particularly those derived from arachidonic acid, can influence mast cell activation and degranulation, which are central to both AR and CSU pathogenesis. The observed upregulation of fatty acid elongation pathways specifically in AR_CSU patients suggests a potential mechanism for the increased symptom severity and chronicity in these patients.

Linoleic Acid Metabolism plays a pivotal role in allergic inflammation through its metabolites (17, 19), particularly hydroxyoctadecadienoic acids (HODEs) (20), which mediate inflammatory responses. The pathway’s significance is manifested through multiple mechanisms, including the modulation of inflammatory mediator production, influence on immune cell function, and impact on epithelial tissue barrier function (21). In allergic rhinitis and other allergic conditions, disrupted fatty acid metabolism has been associated with enhanced inflammatory responses and modified immune responses (19). Metabolic pathways involving fatty acids, including linoleic acid derivatives, contribute to inflammatory mediator release and vascular changes, suggesting their potential role in both AR and CSU pathogenesis (22).

Arachidonic Acid Metabolism, as a central pathway in the production of eicosanoids (18), including prostaglandins and leukotrienes, its differential regulation may explain the distinct inflammatory profiles of AR and AR_CSU. These eicosanoids are key mediators of allergic responses, and their altered production could underlie the more severe or complex symptoms observed in AR_CSU. What’s more, the upregulation of fatty acid elongation and metabolism in AR_CSU, contrasted with their downregulation in AR, suggests divergent inflammatory pathways. This highlights the potential of lipid metabolism as a therapeutic target for modulating inflammation in these conditions.

While our study identified several promising biomarkers, particularly Fasciculic acid C and Biotin-XX-Hydrazide with AUC values around 0.8, we acknowledge the need for external validation in larger and more diverse cohorts. Previous metabolomic studies in allergic diseases have demonstrated significant ethnic variations in metabolic profiles. For instance, Ma et al. reported specific serum metabolomic signatures in Chinese populations with allergic rhinitis (8). The validation of these biomarkers should consider several key aspects. Geographic and ethnic diversity: Studies have shown that metabolomic profiles can vary significantly between different ethnic groups, as highlighted in the work by Kan et al. on ethnic differences in metabolic responses (23). Environmental factors: Local environmental conditions and dietary habits can influence metabolic signatures, as highlighted in comprehensive reviews by Agache and Akdis (4). Disease heterogeneity: The complex nature of both AR and CSU suggests potential subtype-specific metabolic patterns, which aligns with findings from Altrichter et al. (24).

Several limitations of our study should be acknowledged. First, the relatively small sample size, particularly in the AR_CSU group (n=14), may limit the generalizability of our findings. Second, the cross-sectional nature of the study prevents us from establishing causal relationships or temporal changes in metabolic profiles. A significant limitation of our study is the absence of an independent validation cohort, which increases the risk of batch effects and overfitting. Future studies should include larger, independent cohorts to validate these metabolomic signatures and ensure reproducibility. Despite these limitations, our findings suggest promising clinical applications. We propose a translational framework focusing on metabolomic biomarkers (**Supplementary File 3**-**Supplementary Figure S1**), for AR and AR_CSU diagnosis and management. These metabolic signatures could serve as diagnostic tools to differentiate between AR and AR_CSU, enabling early identification of high-risk patients. Additionally, our pathway analyses suggest potential therapeutic targets through metabolic modulation. Future longitudinal studies with larger cohorts will be necessary to validate these findings and establish standardized protocols for their clinical implementation, particularly in the context of personalized treatment approaches.

In conclusion, our study provides novel insights into the metabolic differences between AR and AR_CSU, identifying potential biomarkers and pathways that could inform both diagnostic and therapeutic strategies. These findings contribute to our understanding of the complex pathophysiology of allergic diseases and may lead to more personalized treatment approaches in the future.

## Data Availability

The original contributions presented in the study are included in the article/**Supplementary Material**. Further inquiries can be directed to the corresponding authors.

## References

[1] Wang XD Zheng M Lou HF Wang CS Zhang Y Bo MY. An increased prevalence of self-reported allergic rhinitis in major Chinese cities from 2005 to 2011. Allergy. (2016) 71:1170–80. doi: 10.1111/all.2016.71.issue-8 PMC5074323 26948849

[2] Zheng M Wang X Bo M Wang K Zhao Y He F. Prevalence of allergic rhinitis among adults in urban and rural areas of China: a population-based cross-sectional survey. Allergy Asthma Immunol Res. (2015) 7:148–57. doi: 10.4168/aair.2015.7.2.148 PMC4341336 25729622

[3] Zuberbier T Aberer W Asero R Abdul Latiff AH Baker D Ballmer-Weber B. The EAACI/GA²LEN/EDF/WAO guideline for the definition, classification, diagnosis and management of urticaria. Allergy. (2018) 73:1393–414. doi: 10.1111/all.2018.73.issue-7 29336054

[4] Agache I Akdis CA. Precision medicine and phenotypes, endotypes, genotypes, regiotypes, and theratypes of allergic diseases. J Clin Invest. (2019) 129:1493–503. doi: 10.1172/JCI124611 PMC6436902 30855278

[5] Galli SJ Tsai M Piliponsky AM. The development of allergic inflammation. Nature. (2008) 454:445–54. doi: 10.1038/nature07204 PMC3573758 18650915

[6] Xie S Jiang S Zhang H Wang F Liu Y She Y. Prediction of sublingual immunotherapy efficacy in allergic rhinitis by serum metabolomics analysis. Int Immunopharmacol. (2021) 90:107211. doi: 10.1016/j.intimp.2020.107211 33271394

[7] Kolmert J Gómez C Balgoma D Sjödin M Bood J Konradsen JR. Urinary leukotriene E(4) and prostaglandin D(2) metabolites increase in adult and childhood severe asthma characterized by type 2 inflammation. A clinical observational study. Am J Respir Crit Care Med. (2021) 203:37–53. doi: 10.1164/rccm.201909-1869OC 32667261 PMC7781128

[8] Ma GC Wang TS Wang J Ma ZJ Pu SB. Serum metabolomics study of patients with allergic rhinitis. BioMed Chromatogr. (2020) 34:e4739. doi: 10.1002/bmc.v34.3 31692004

[9] Wang X Yi W He L Luo S Wang J Jiang L. Abnormalities in gut microbiota and metabolism in patients with chronic spontaneous urticaria. Front Immunol. (2021) 12:691304. doi: 10.3389/fimmu.2021.691304 34721374 PMC8554312

[10] Lu T Chen Y Guo Y Sun J Shen W Yuan M. Altered gut microbiota diversity and composition in chronic urticaria. Dis Markers. (2019) 2019:6417471. doi: 10.1155/2019/6417471 31827639 PMC6881578

[11] Brożek JL Bousquet J Agache I Agarwal A Bachert C Bosnic-Anticevich S. Allergic Rhinitis and its Impact on Asthma (ARIA) guidelines-2016 revision. J Allergy Clin Immunol. (2017) 140:950–8. doi: 10.1016/j.jaci.2017.03.050 28602936

[12] Demoly P Bousquet PJ Mesbah K Bousquet J Devillier P. Visual analogue scale in patients treated for allergic rhinitis: an observational prospective study in primary care: asthma and rhinitis. Clin Exp Allergy. (2013) 43:881–8. doi: 10.1111/cea.2013.43.issue-8 23889242

[13] Hawro T Ohanyan T Schoepke N Metz M Peveling-Oberhag A Staubach P. The urticaria activity score-validity, reliability, and responsiveness. J Allergy Clin Immunol Pract. (2018) 6:1185–1190.e1. doi: 10.1016/j.jaip.2017.10.001 29128337

[14] Bousquet J Bousquet J Agache I Agarwal A Bachert C Bosnic-Anticevich S. Allergic Rhinitis and its Impact on Asthma (ARIA) 2008 update (in collaboration with the World Health Organization, GA(2)LEN and AllerGen). Allergy. (2008) 63 Suppl 86:8–160. doi: 10.1111/j.1398-9995.2007.01620.x 18331513

[15] Takahashi A Khaltaev N Cruz AA Denburg J Fokkens WJ Togias A. Fasciculic acids A, B and C as calmodulin antagonists from the mushroom Naematoloma fasciculare. Chem Pharm Bull (Tokyo). (1989) 37:3247–50. doi: 10.1248/cpb.37.3247 2632070

[16] Agrawal S Agrawal A Said HM. Biotin deficiency enhances the inflammatory response of human dendritic cells. Am J Physiol Cell Physiol. (2016) 311:C386–91. doi: 10.1152/ajpcell.00141.2016 PMC5129763 27413170

[17] Miyata J Arita M. Role of omega-3 fatty acids and their metabolites in asthma and allergic diseases. Allergol Int. (2015) 64:27–34. doi: 10.1016/j.alit.2014.08.003 25572556

[18] Dennis EA Norris PC. Eicosanoid storm in infection and inflammation. Nat Rev Immunol. (2015) 15:511–23. doi: 10.1038/nri3859 PMC4606863 26139350

[19] Yuan Y Norris PC. Airway microbiome and serum metabolomics analysis identify differential candidate biomarkers in allergic rhinitis. Front Immunol. (2021) 12:771136. doi: 10.3389/fimmu.2021.771136 35069544 PMC8766840

[20] Mabalirajan U Wang C Wang G Guo X Jiang S Zuo X. Linoleic acid metabolite drives severe asthma by causing airway epithelial injury. Sci Rep. (2013) 3:1349. doi: 10.1038/srep01349 23443229 PMC3583002

[21] Naughten S Rehman R Ahmad T Kumar S Singh S Leishangthem GD. The re-emerging role of linoleic acid in paediatric asthma. Eur Respir Rev. (2023) 32(170):230063. doi: 10.1183/16000617.0063-2023 37914192 PMC10618909

[22] Ramsden CE Ecklu-Mensah G Constantino G Quaranta A Schulkers Escalante K Bai-Tong S. A systems approach for discovering linoleic acid derivatives that potentially mediate pain and itch. Sci Signal. (2017) 10(493):eaal5241. doi: 10.1126/scisignal.aal5241 28831021 PMC5805383

[23] Stefan N Domenichiello AF Yuan ZX Sapio MR Keyes GS Mishra SK. Exaggerated insulin secretion in Pima Indians and African-Americans but higher insulin resistance in Pima Indians compared to African-Americans and Caucasians. Diabetes Med. (2004) 21:1090–5. doi: 10.1111/j.1464-5491.2004.01290.x 15384955

[24] Altrichter S Stumvoll M Weyer C Bogardus C Tataranni PA Pratley RE. Serum IgE autoantibodies target keratinocytes in patients with atopic dermatitis. J Invest Dermatol. (2008) 128:2232–9. doi: 10.1038/jid.2008.80 18480840

