# Supplementary figures and images for: Distinct metabolomic signatures in allergic rhinitis with concurrent chronic spontaneous urticaria: an untargeted metabolomics analysis reveals novel biomarkers and pathway alterations
Source: Front Immunol. 2025 Jun 10;16:1555664. doi: 10.3389/fimmu.2025.1555664 (PMC12185459; doi:10.3389/fimmu.2025.1555664)

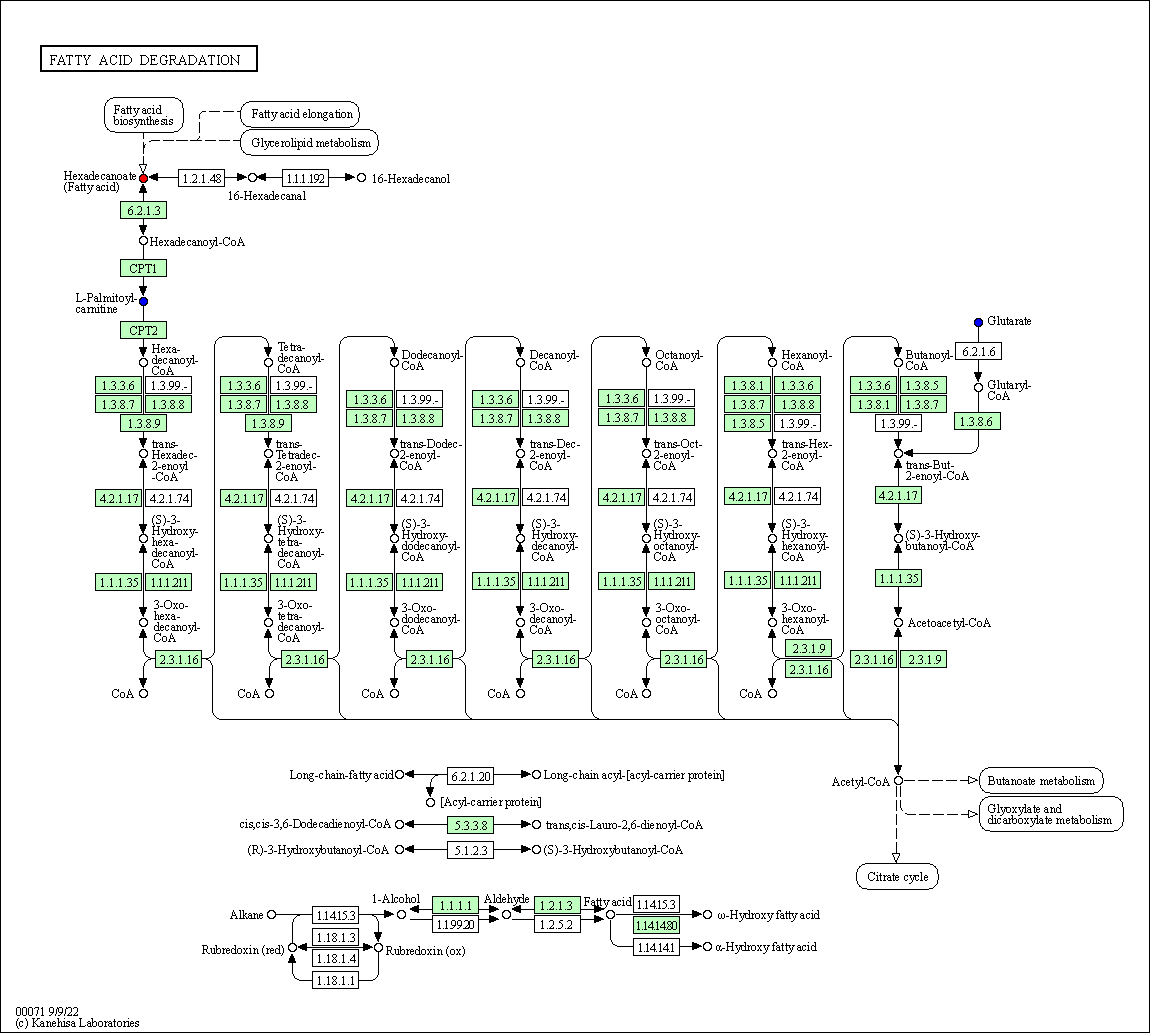

Supplement: Supplementary file 3 [file SupplementaryFile3.zip › 20241027-Supplementary files 4-Figure4-pathway-36σ╝áσ¢╛/ko00071.png]

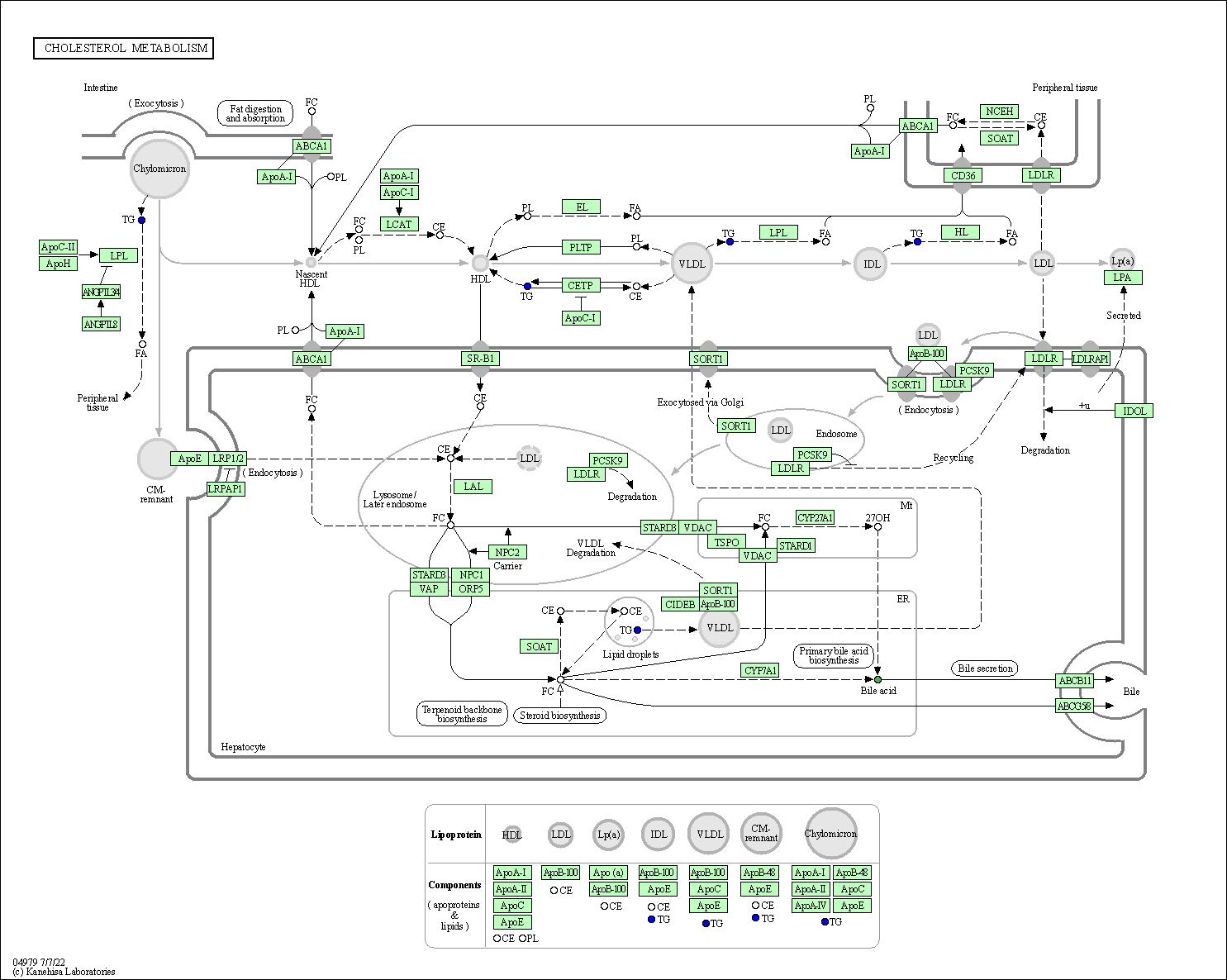

Supplement: Supplementary file 3 [file SupplementaryFile3.zip › 20241027-Supplementary files 4-Figure4-pathway-36σ╝áσ¢╛/ko04979.png]

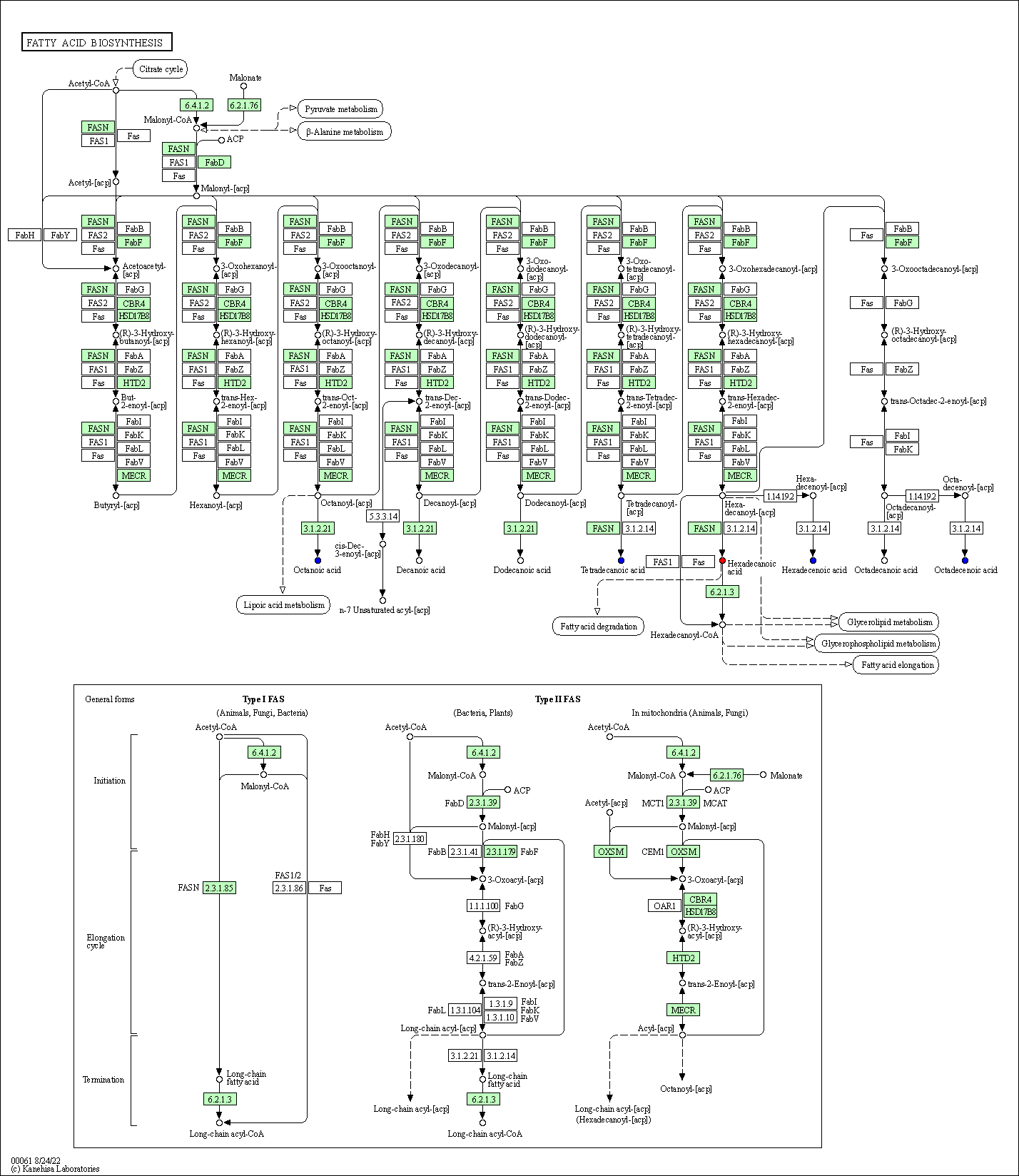

Supplement: Supplementary file 3 [file SupplementaryFile3.zip › 20241027-Supplementary files 4-Figure4-pathway-36σ╝áσ¢╛/ko00061.png]

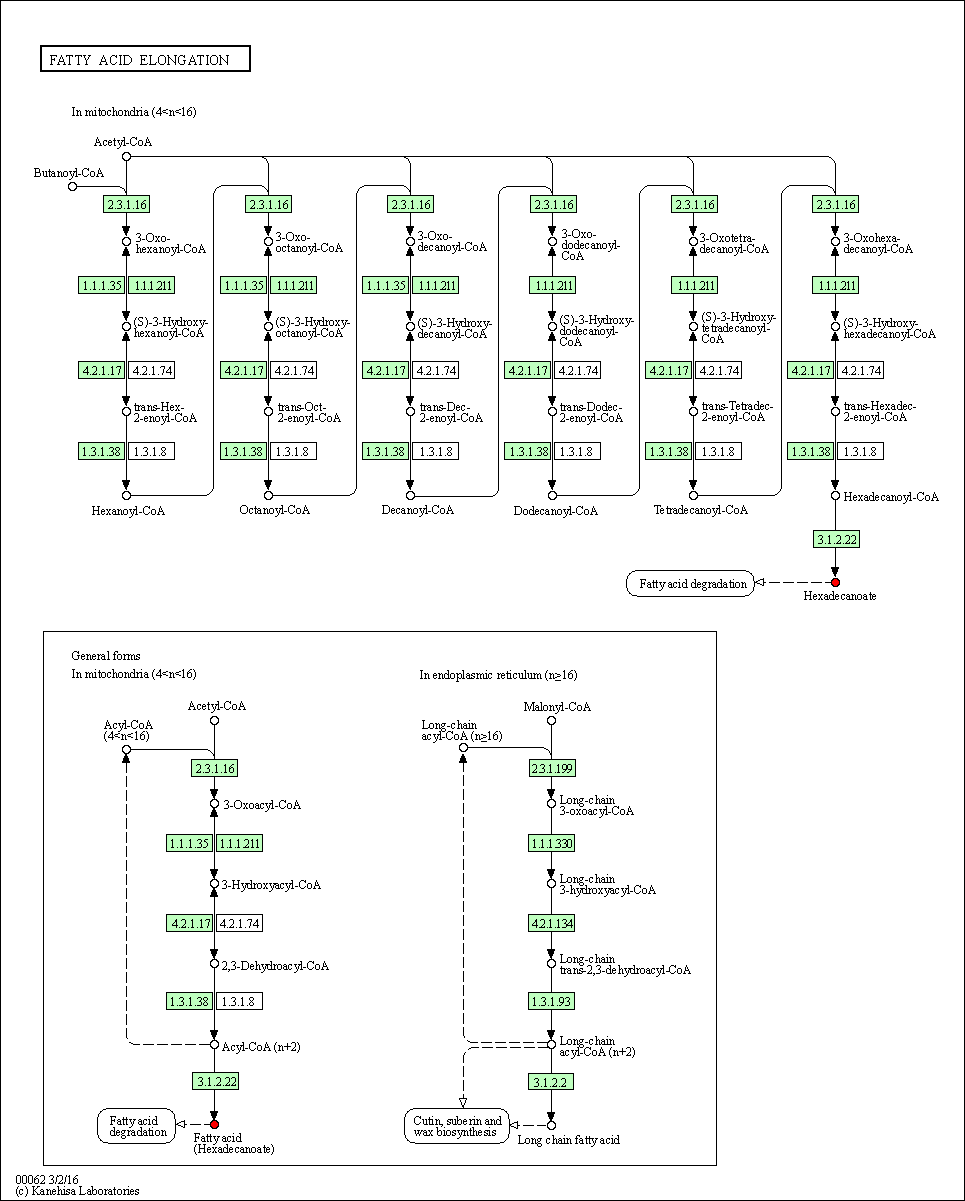

Supplement: Supplementary file 3 [file SupplementaryFile3.zip › 20241027-Supplementary files 4-Figure4-pathway-36σ╝áσ¢╛/ko00062.png]

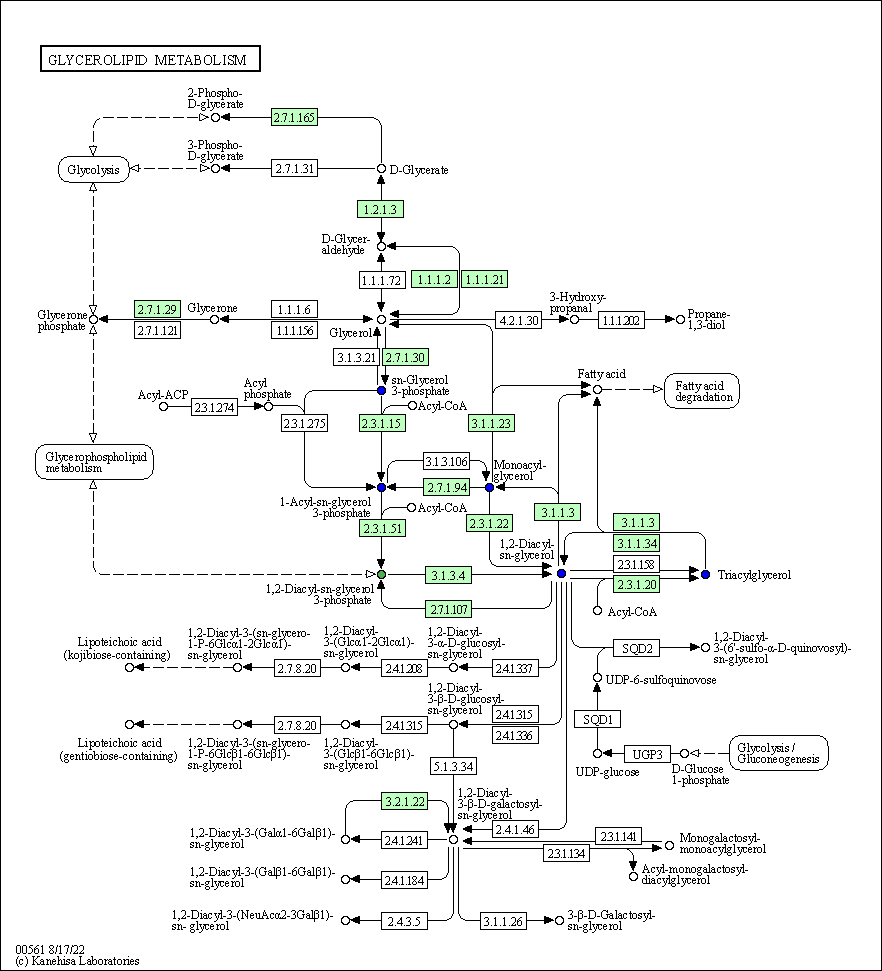

Supplement: Supplementary file 3 [file SupplementaryFile3.zip › 20241027-Supplementary files 4-Figure4-pathway-36σ╝áσ¢╛/ko00561.png]

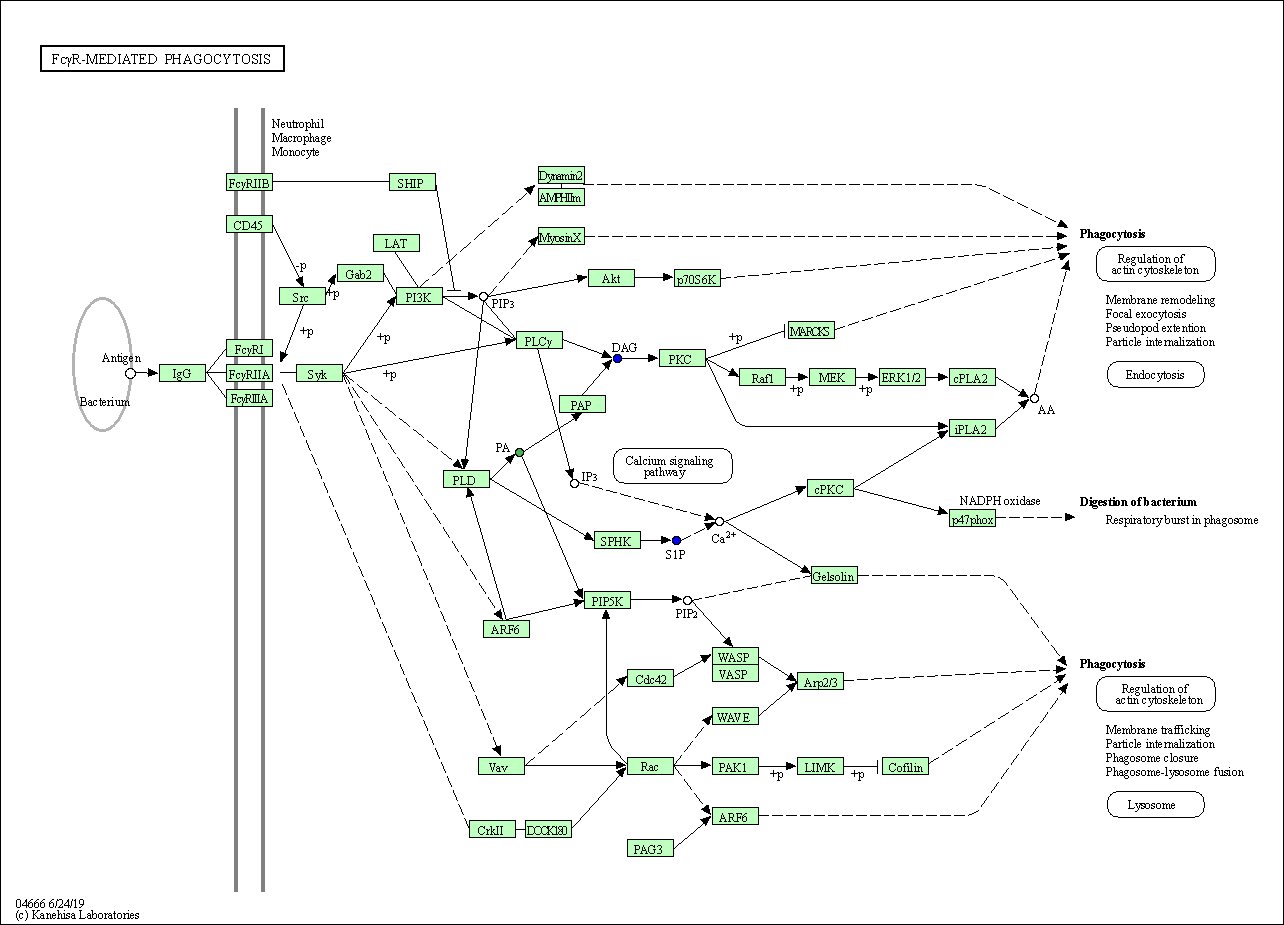

Supplement: Supplementary file 3 [file SupplementaryFile3.zip › 20241027-Supplementary files 4-Figure4-pathway-36σ╝áσ¢╛/ko04666.png]

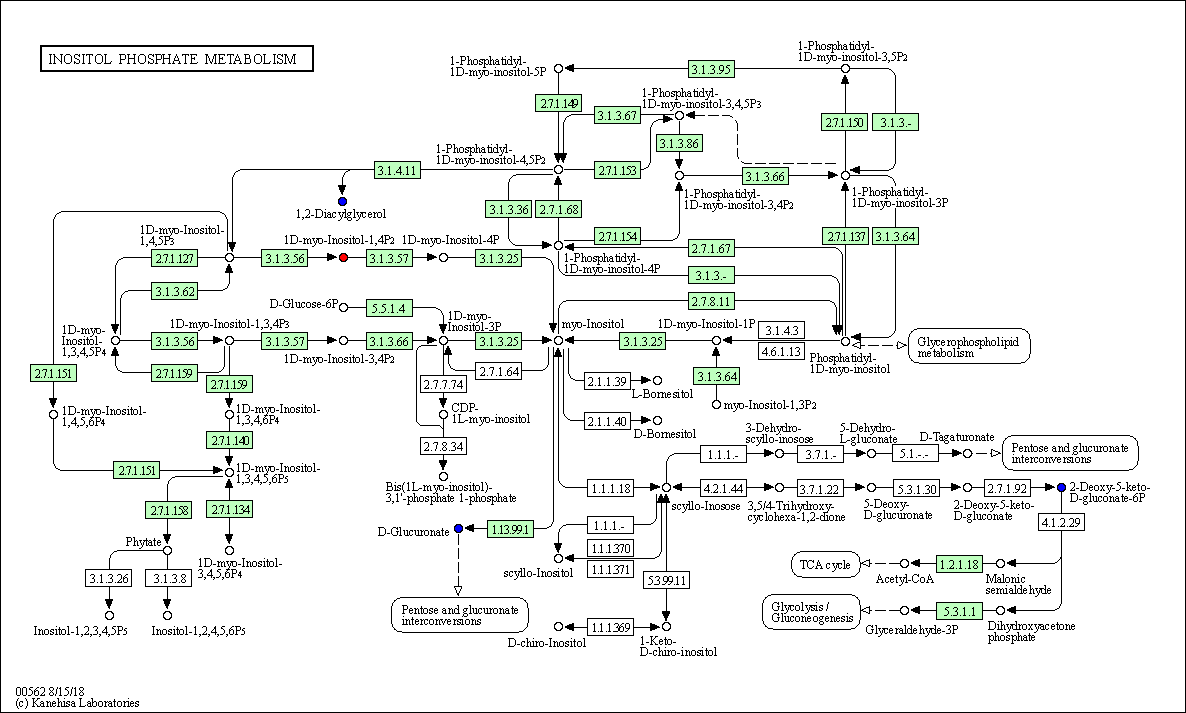

Supplement: Supplementary file 3 [file SupplementaryFile3.zip › 20241027-Supplementary files 4-Figure4-pathway-36σ╝áσ¢╛/ko00562.png]

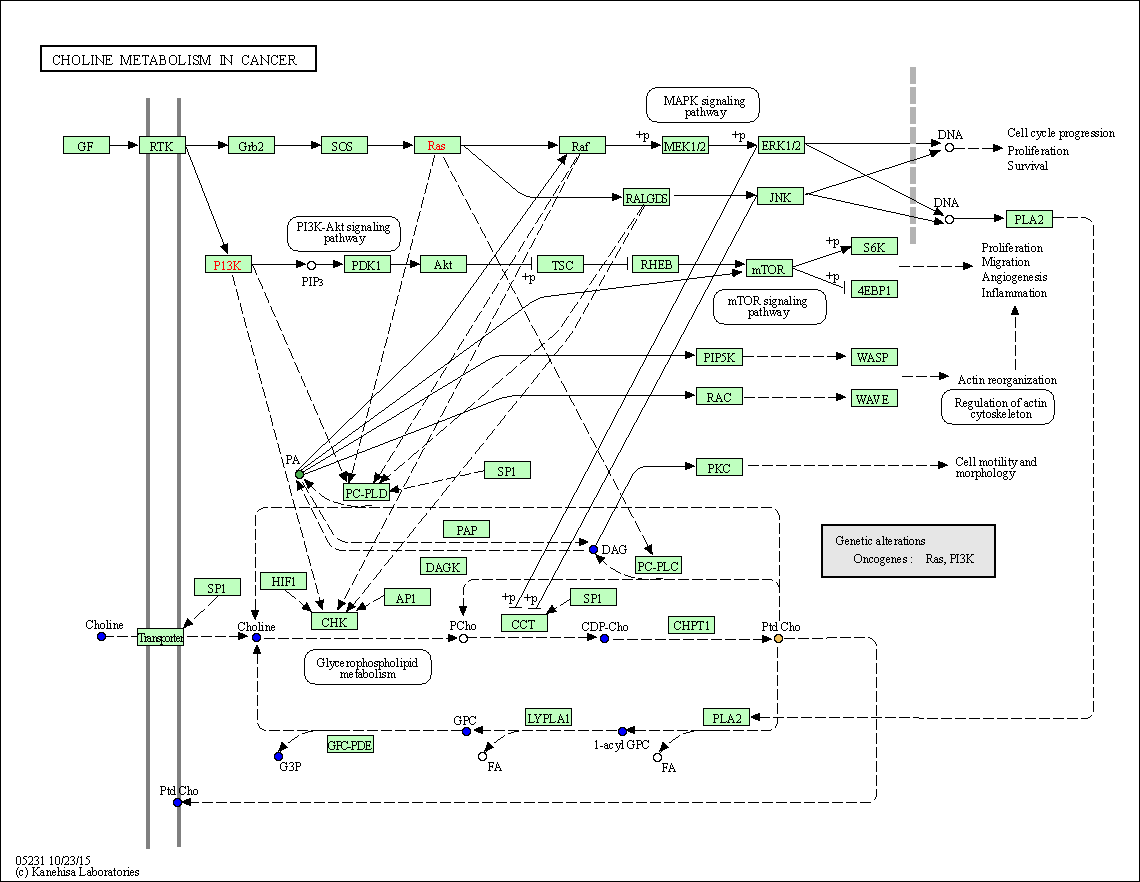

Supplement: Supplementary file 3 [file SupplementaryFile3.zip › 20241027-Supplementary files 4-Figure4-pathway-36σ╝áσ¢╛/ko05231.png]

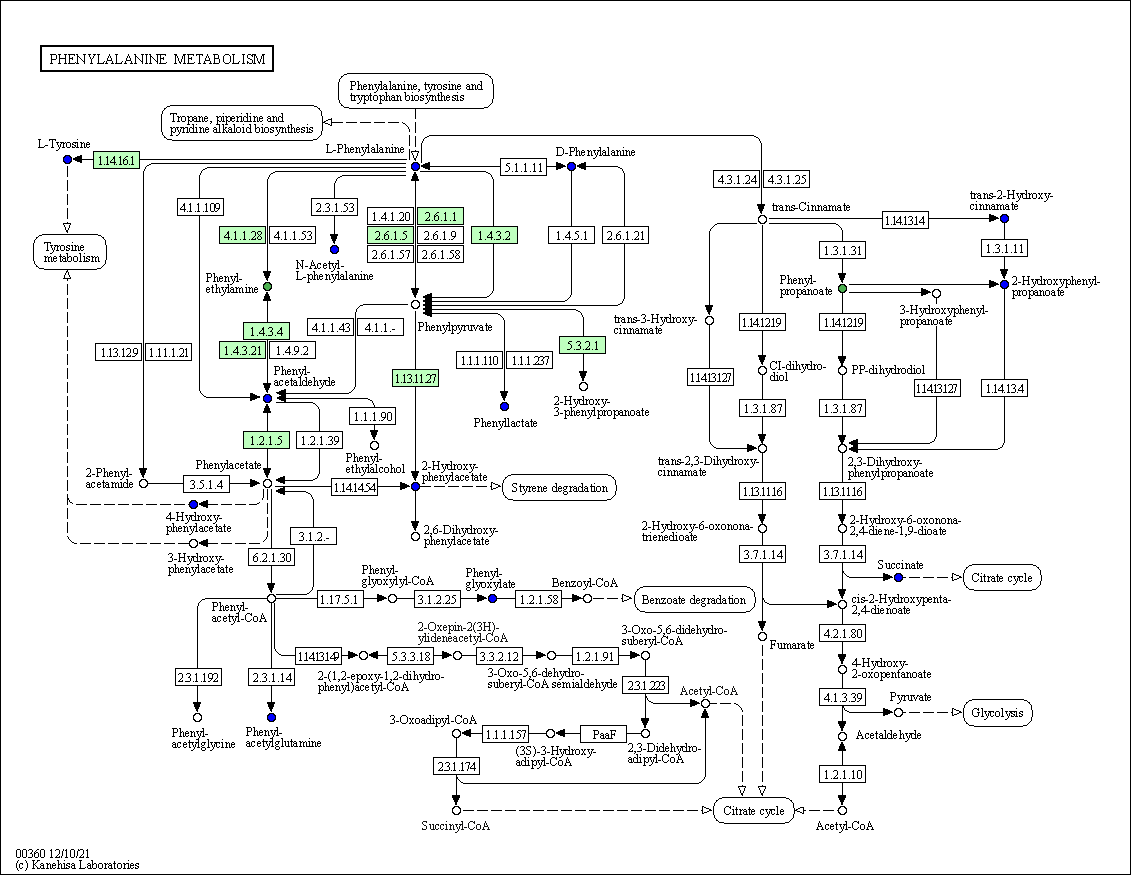

Supplement: Supplementary file 3 [file SupplementaryFile3.zip › 20241027-Supplementary files 4-Figure4-pathway-36σ╝áσ¢╛/ko00360.png]

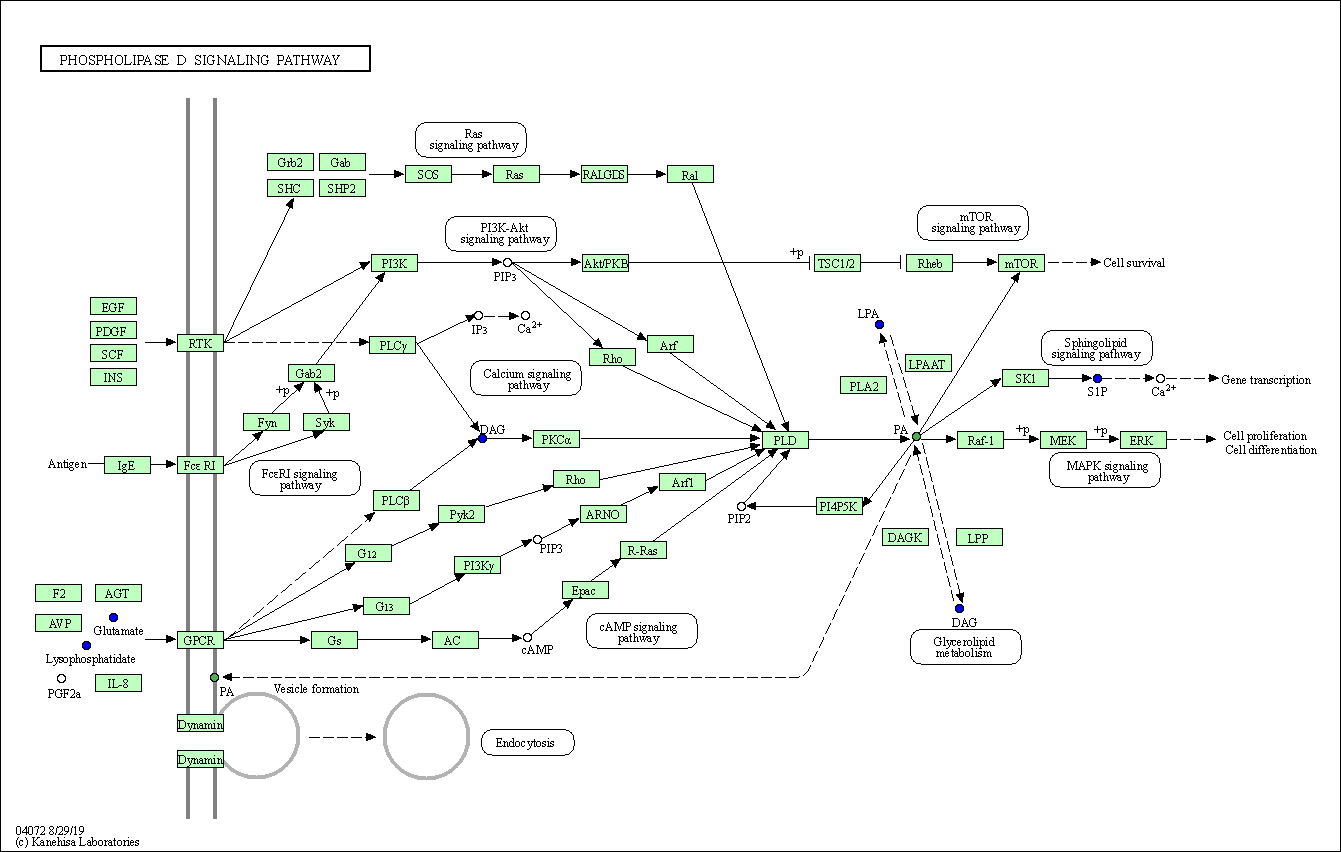

Supplement: Supplementary file 3 [file SupplementaryFile3.zip › 20241027-Supplementary files 4-Figure4-pathway-36σ╝áσ¢╛/ko04072.png]

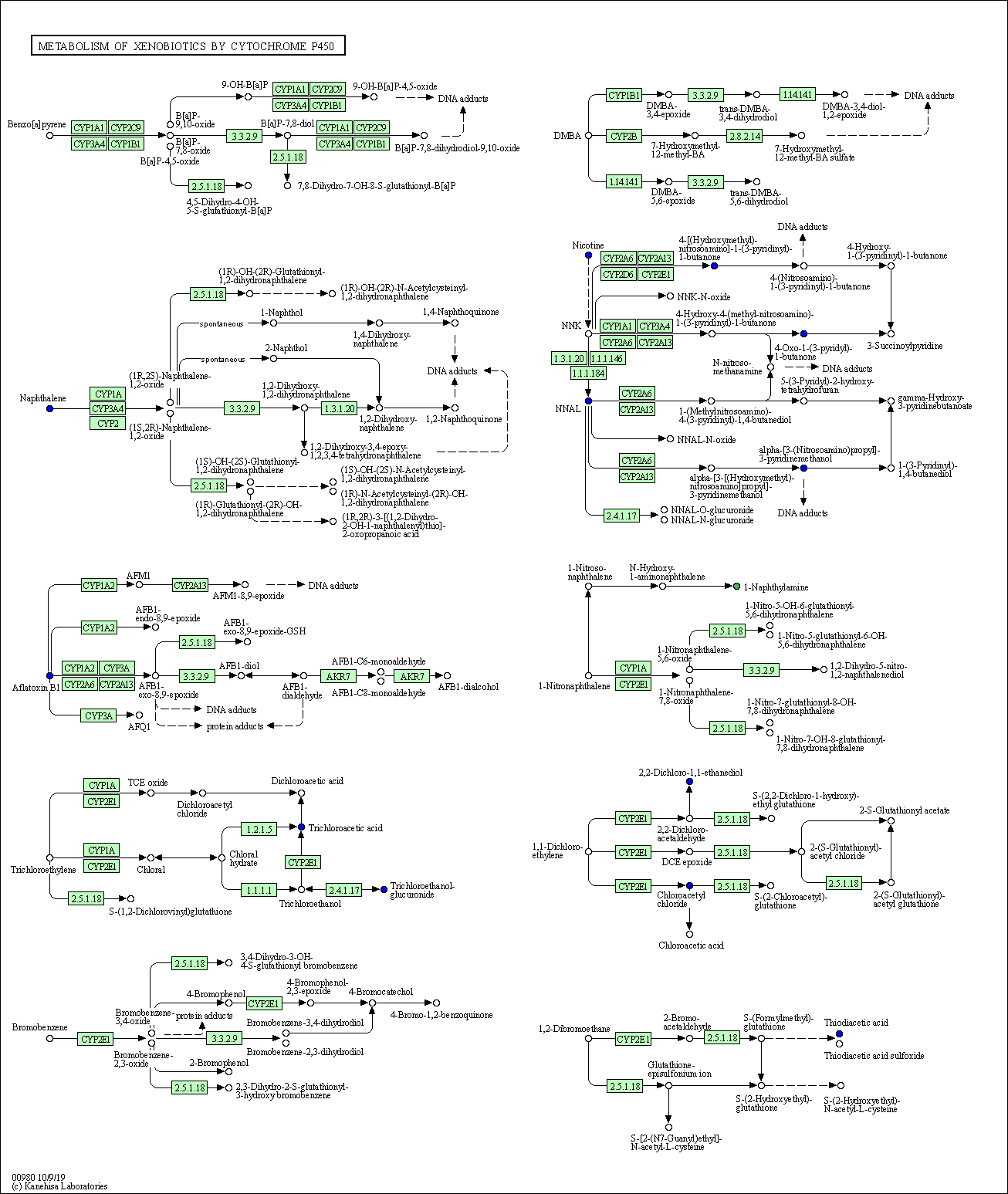

Supplement: Supplementary file 3 [file SupplementaryFile3.zip › 20241027-Supplementary files 4-Figure4-pathway-36σ╝áσ¢╛/ko00980.png]

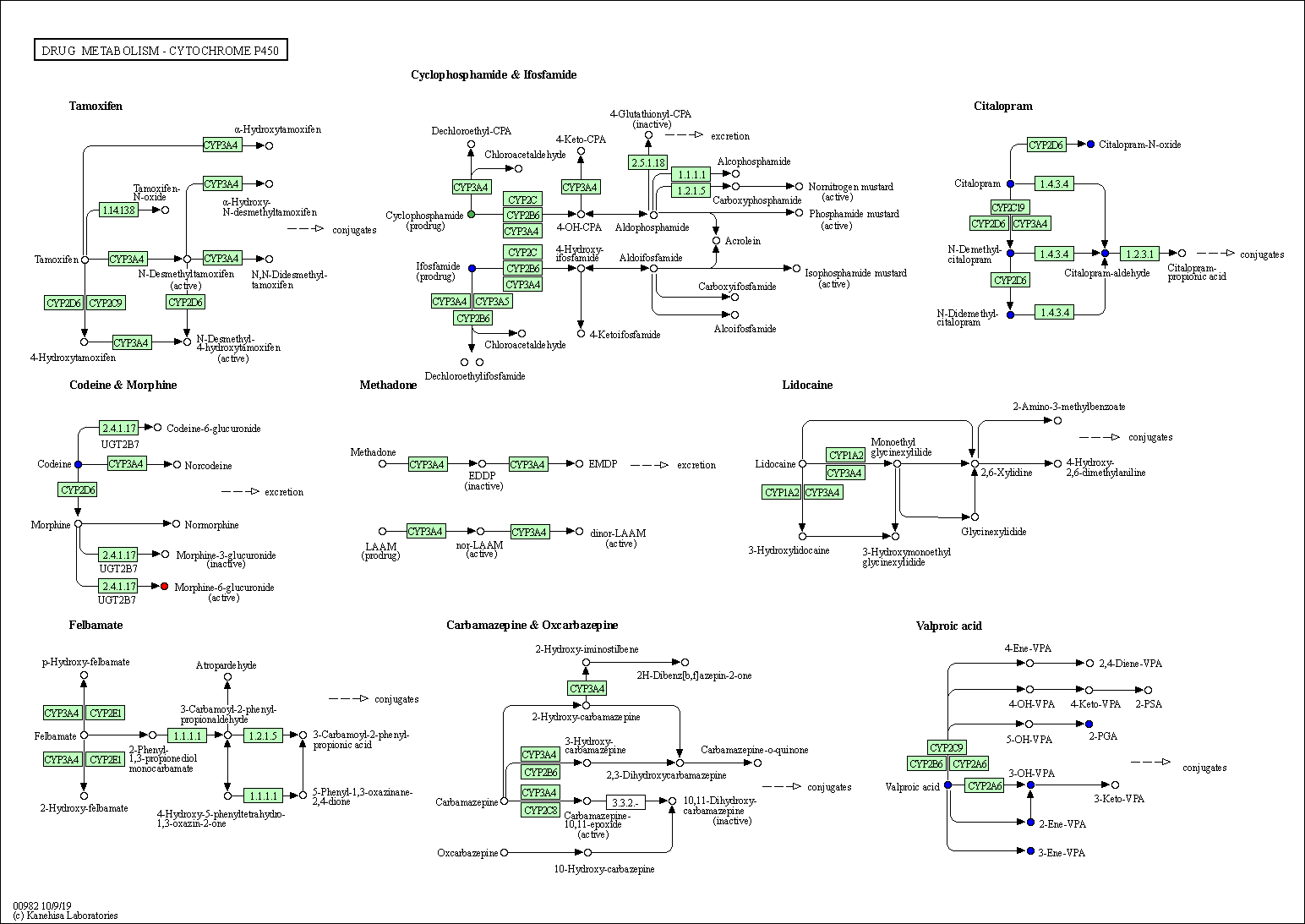

Supplement: Supplementary file 3 [file SupplementaryFile3.zip › 20241027-Supplementary files 4-Figure4-pathway-36σ╝áσ¢╛/ko00982.png]

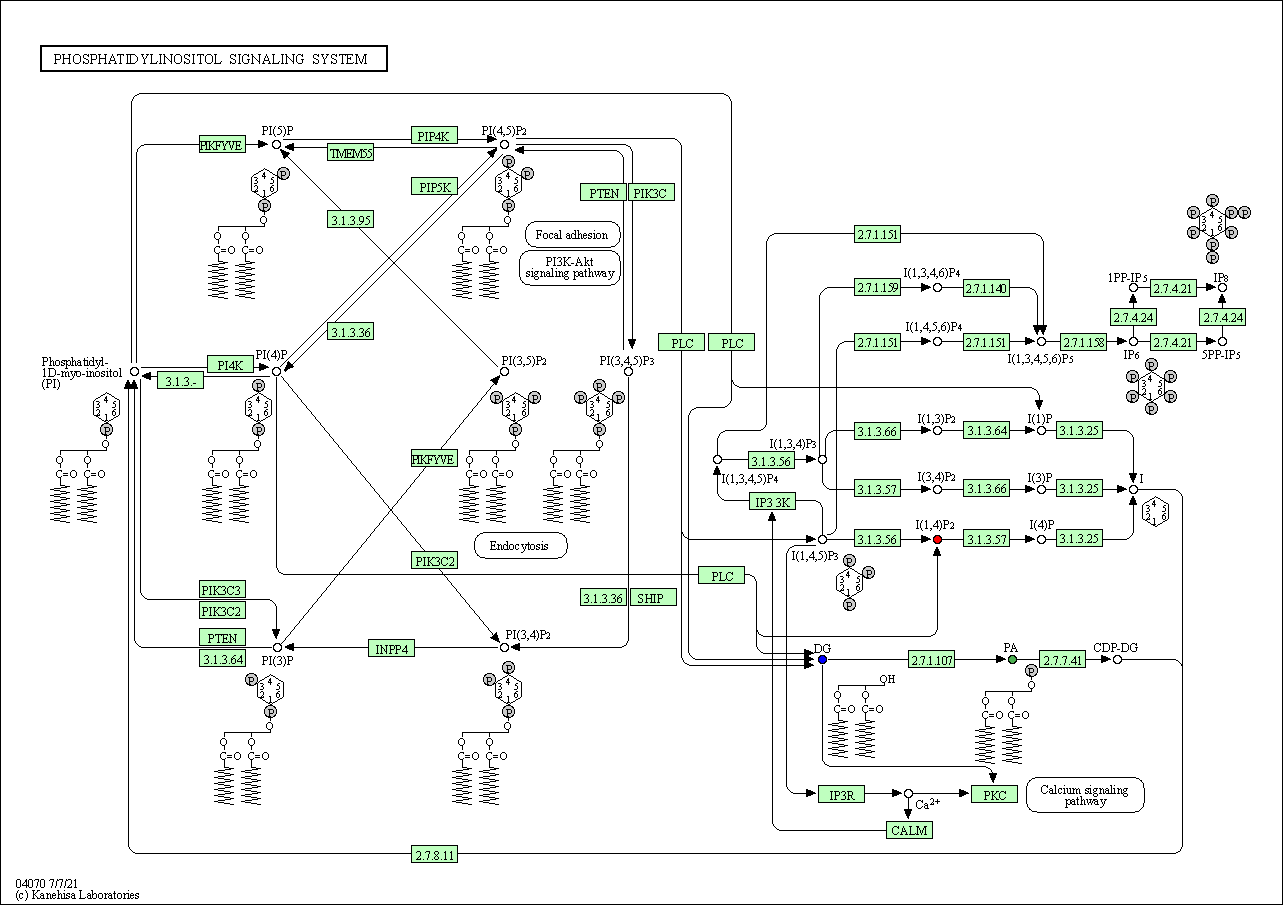

Supplement: Supplementary file 3 [file SupplementaryFile3.zip › 20241027-Supplementary files 4-Figure4-pathway-36σ╝áσ¢╛/ko04070.png]

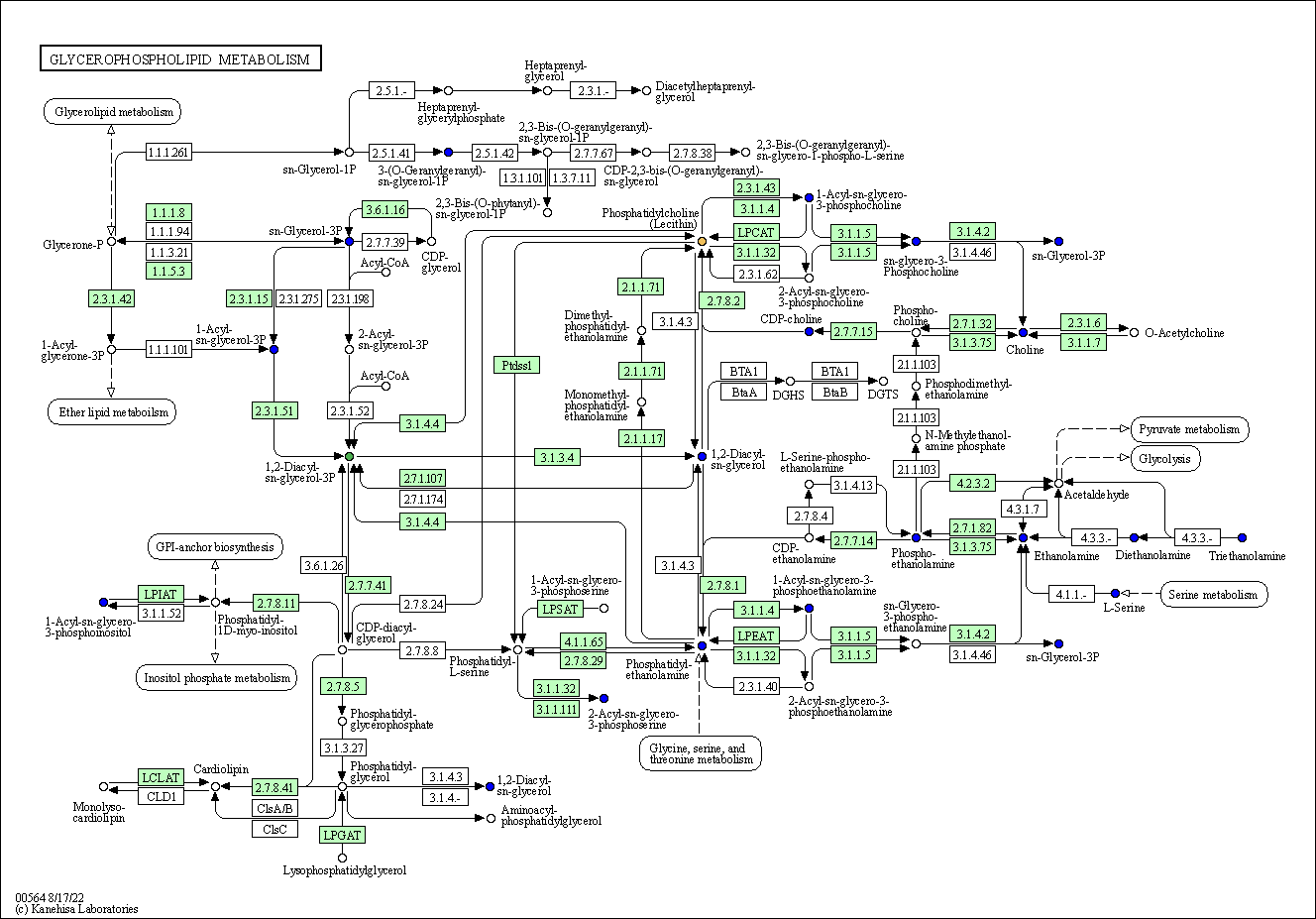

Supplement: Supplementary file 3 [file SupplementaryFile3.zip › 20241027-Supplementary files 4-Figure4-pathway-36σ╝áσ¢╛/ko00564.png]

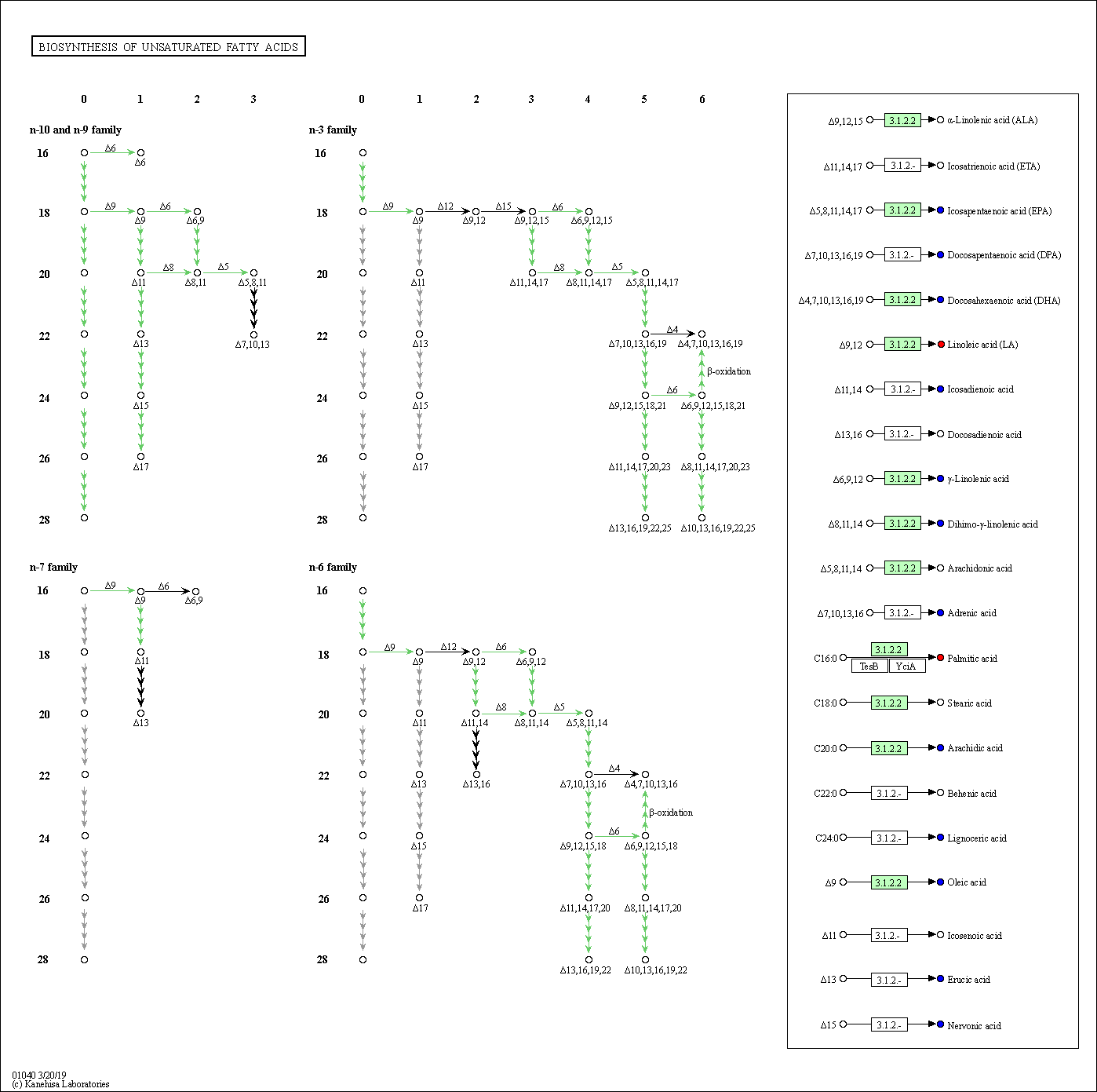

Supplement: Supplementary file 3 [file SupplementaryFile3.zip › 20241027-Supplementary files 4-Figure4-pathway-36σ╝áσ¢╛/ko01040.png]

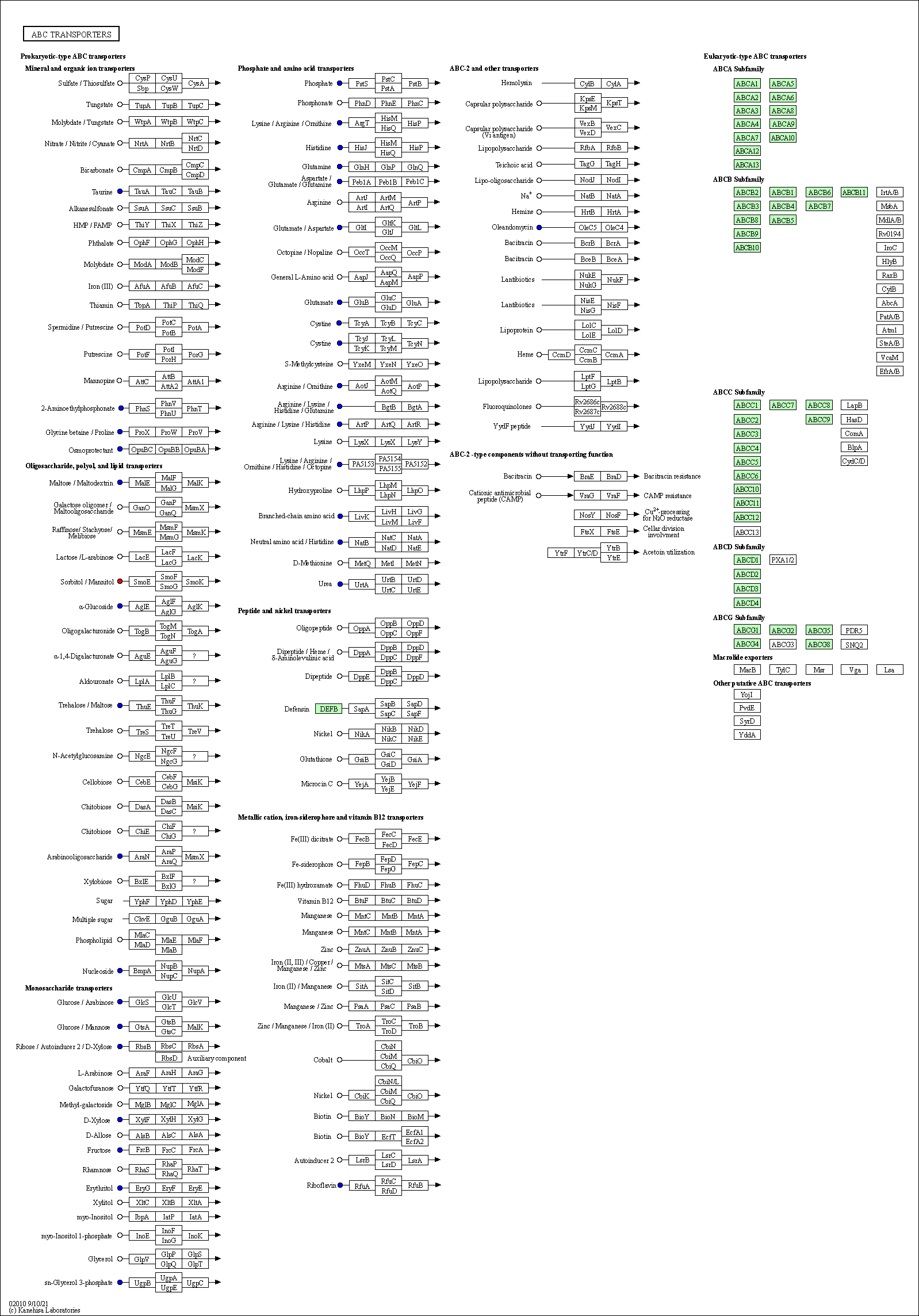

Supplement: Supplementary file 3 [file SupplementaryFile3.zip › 20241027-Supplementary files 4-Figure4-pathway-36σ╝áσ¢╛/ko02010.png]

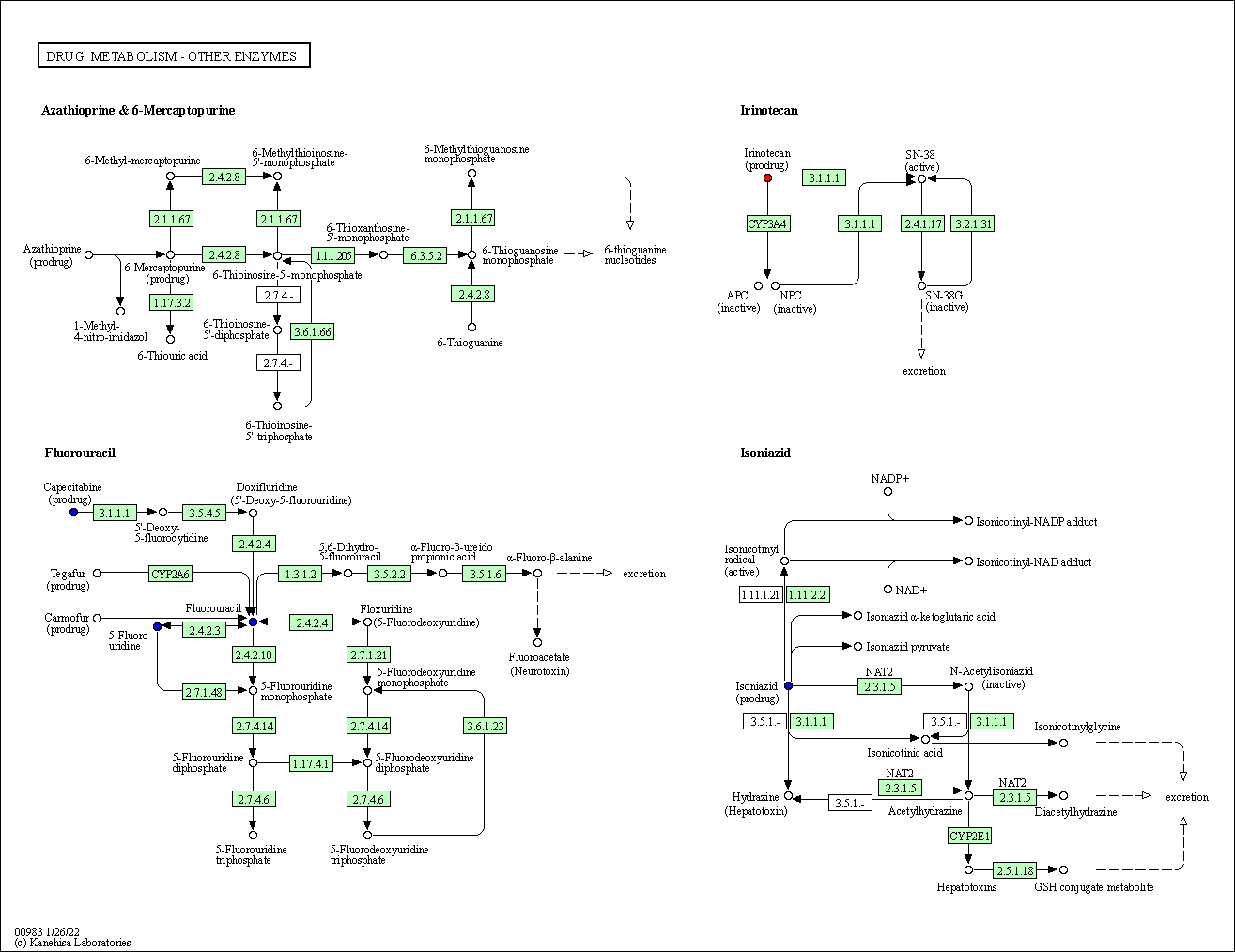

Supplement: Supplementary file 3 [file SupplementaryFile3.zip › 20241027-Supplementary files 4-Figure4-pathway-36σ╝áσ¢╛/ko00983.png]

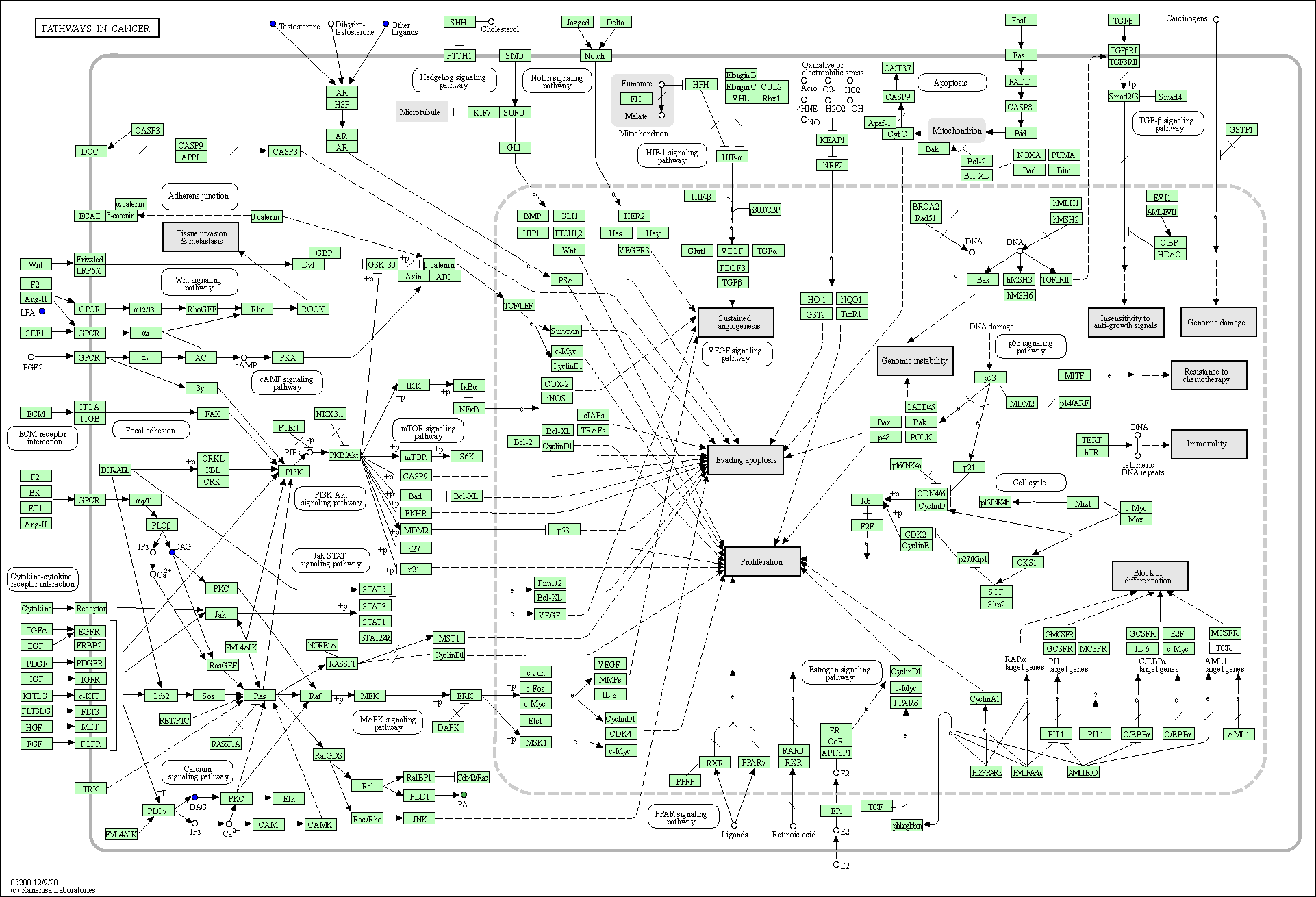

Supplement: Supplementary file 3 [file SupplementaryFile3.zip › 20241027-Supplementary files 4-Figure4-pathway-36σ╝áσ¢╛/ko05200.png]

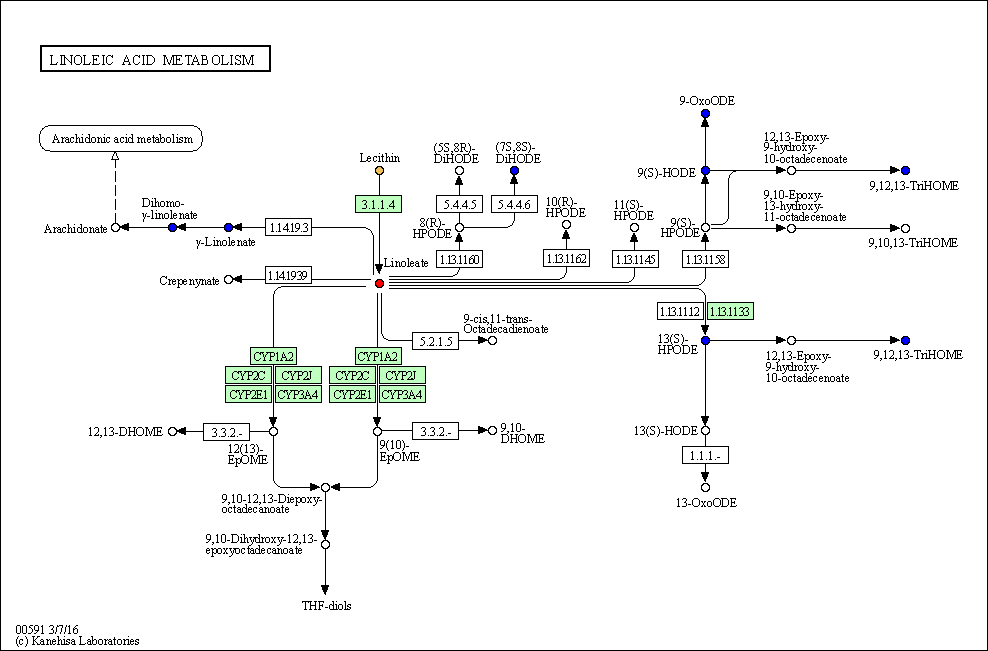

Supplement: Supplementary file 3 [file SupplementaryFile3.zip › 20241027-Supplementary files 4-Figure4-pathway-36σ╝áσ¢╛/ko00591.png]

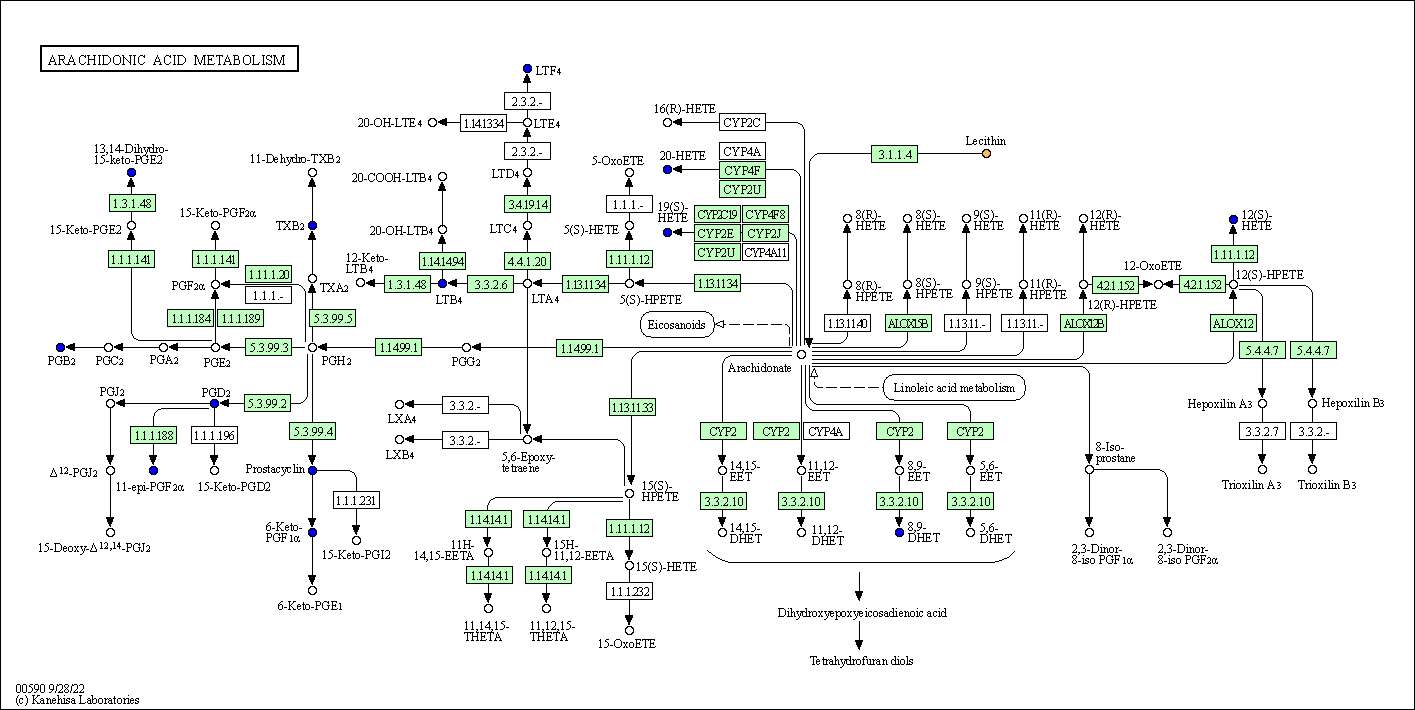

Supplement: Supplementary file 3 [file SupplementaryFile3.zip › 20241027-Supplementary files 4-Figure4-pathway-36σ╝áσ¢╛/ko00590.png]

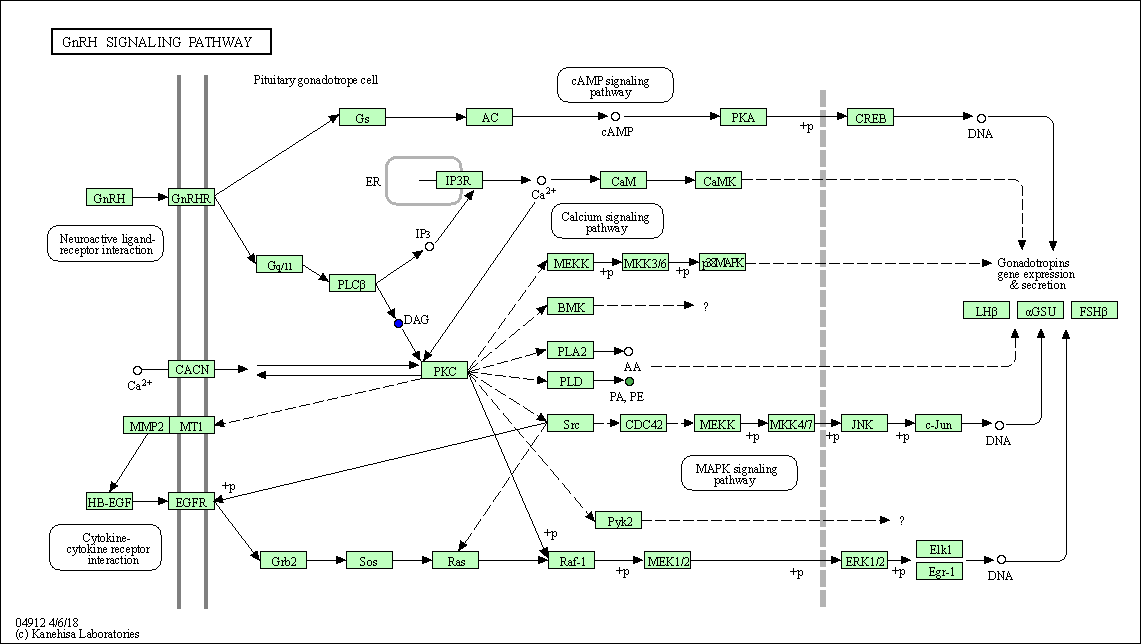

Supplement: Supplementary file 3 [file SupplementaryFile3.zip › 20241027-Supplementary files 4-Figure4-pathway-36σ╝áσ¢╛/ko04912.png]

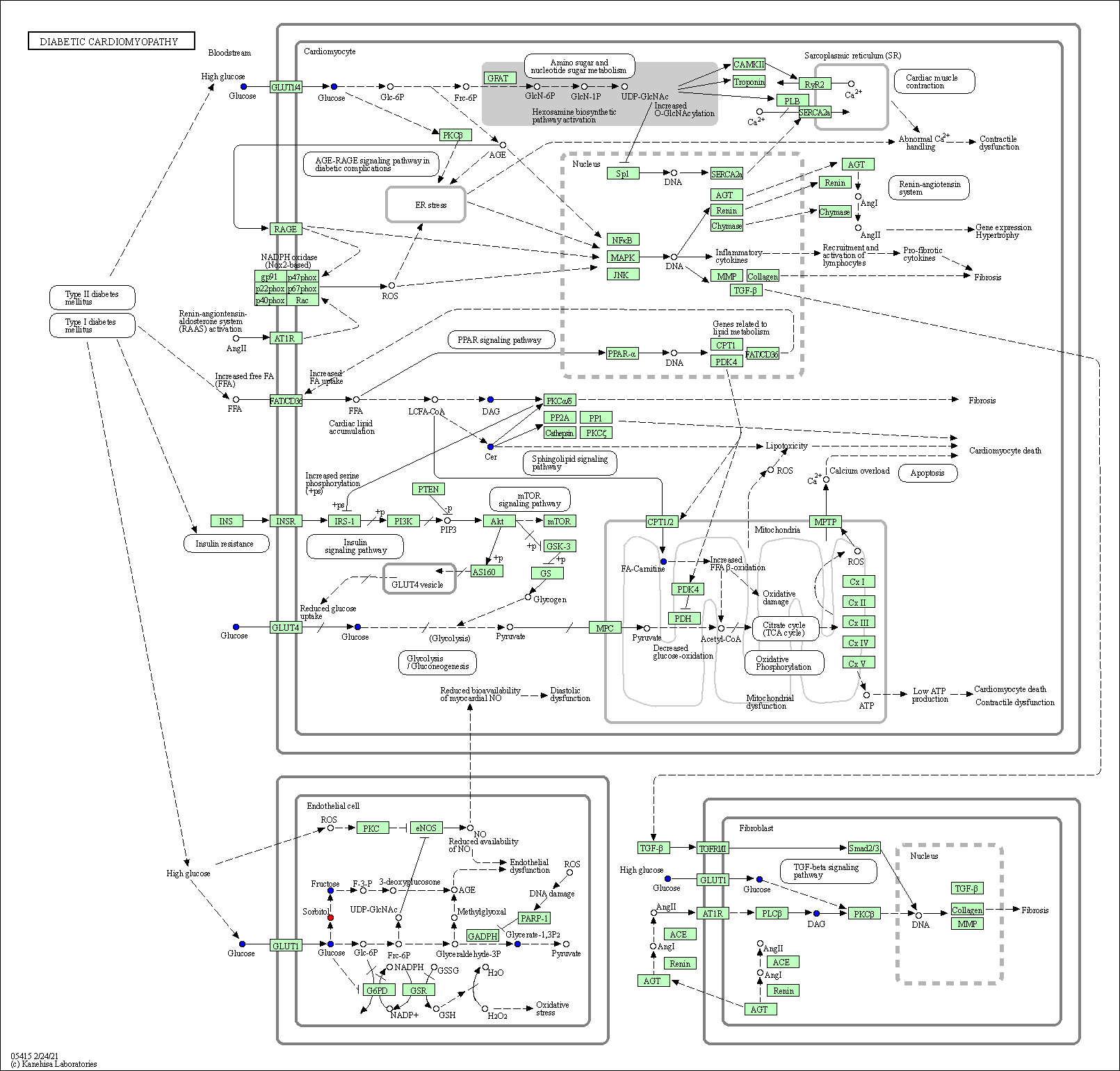

Supplement: Supplementary file 3 [file SupplementaryFile3.zip › 20241027-Supplementary files 4-Figure4-pathway-36σ╝áσ¢╛/ko05415.png]

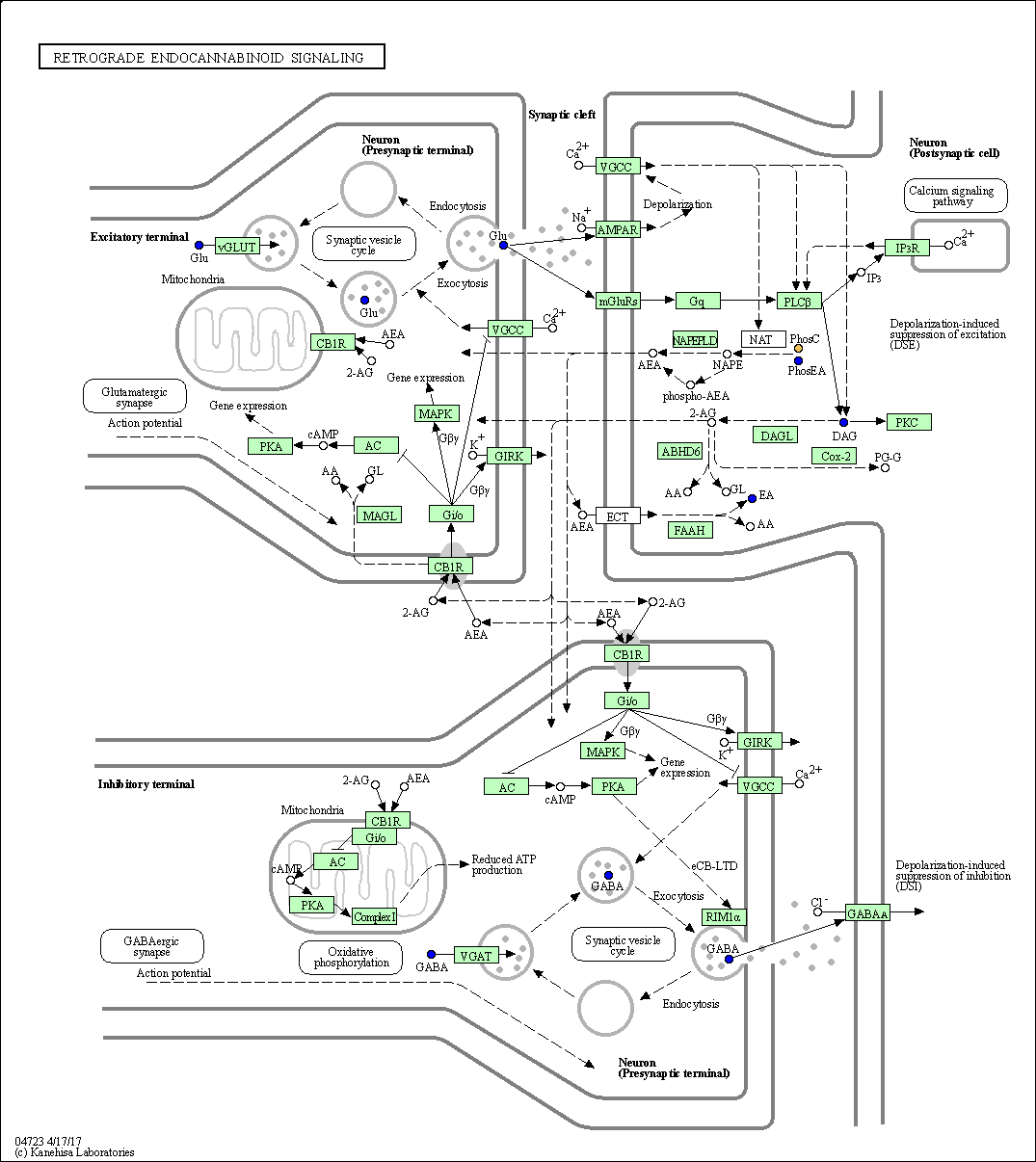

Supplement: Supplementary file 3 [file SupplementaryFile3.zip › 20241027-Supplementary files 4-Figure4-pathway-36σ╝áσ¢╛/ko04723.png]

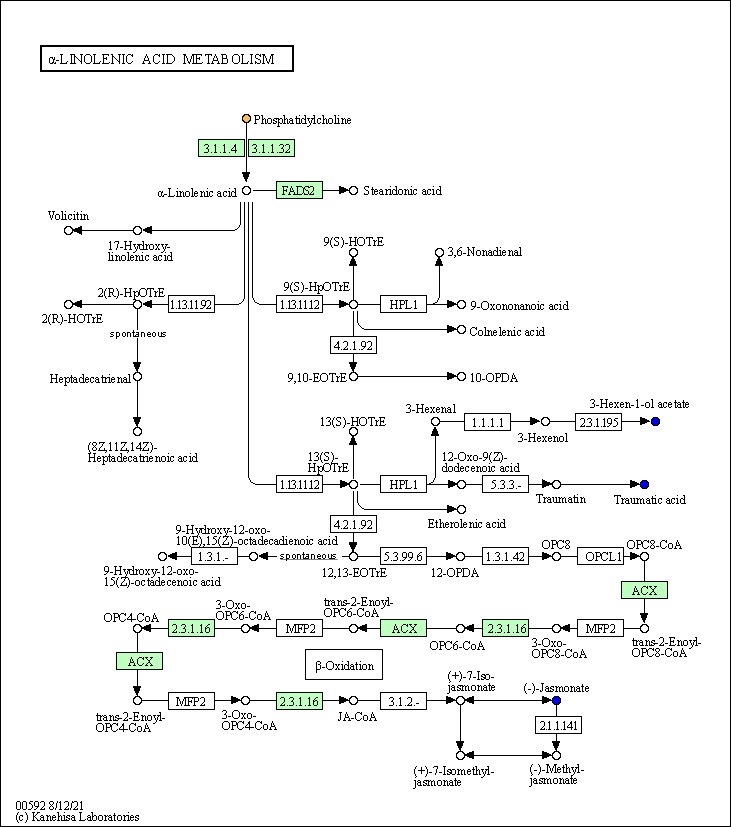

Supplement: Supplementary file 3 [file SupplementaryFile3.zip › 20241027-Supplementary files 4-Figure4-pathway-36σ╝áσ¢╛/ko00592.png]

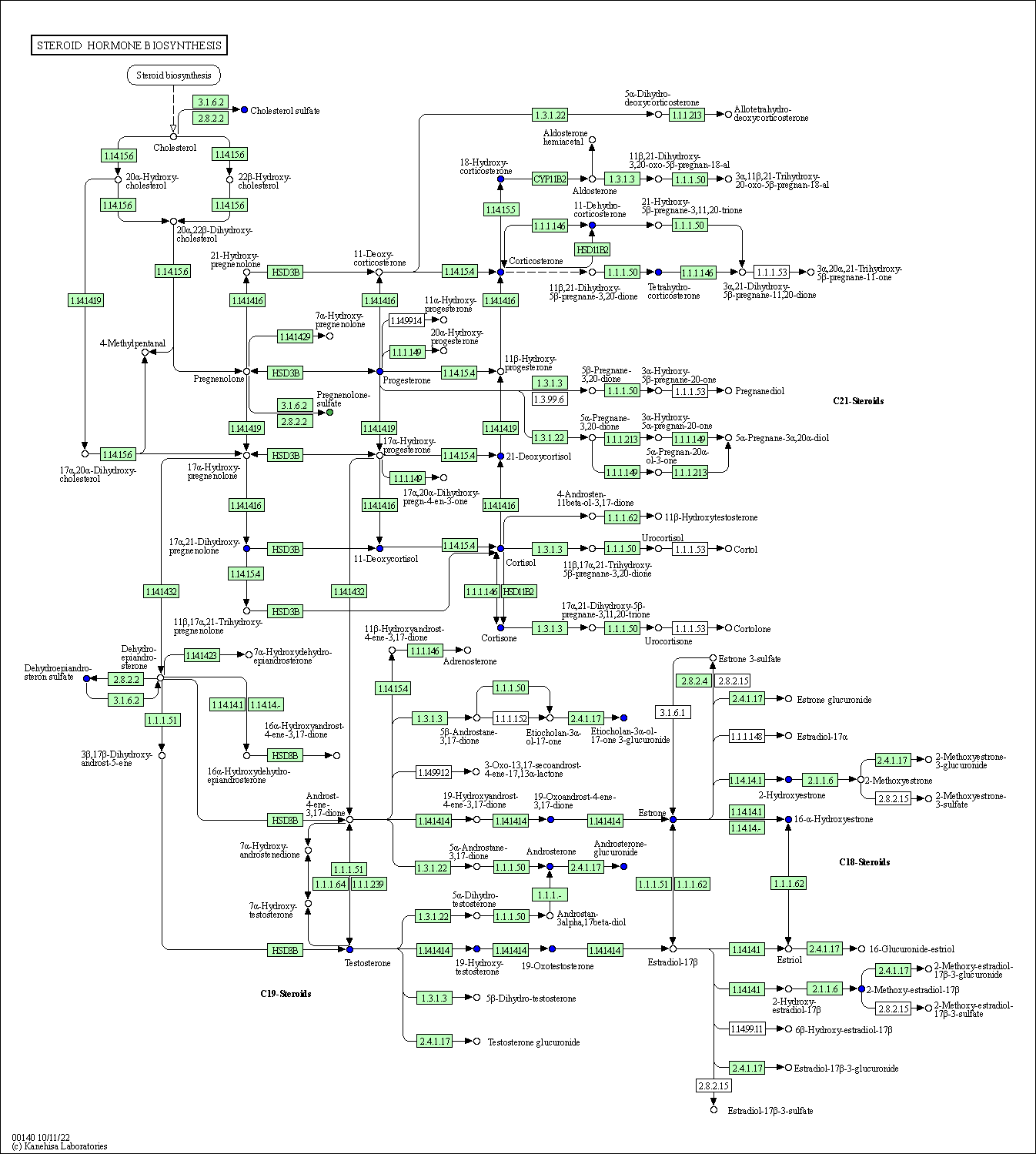

Supplement: Supplementary file 3 [file SupplementaryFile3.zip › 20241027-Supplementary files 4-Figure4-pathway-36σ╝áσ¢╛/ko00140.png]

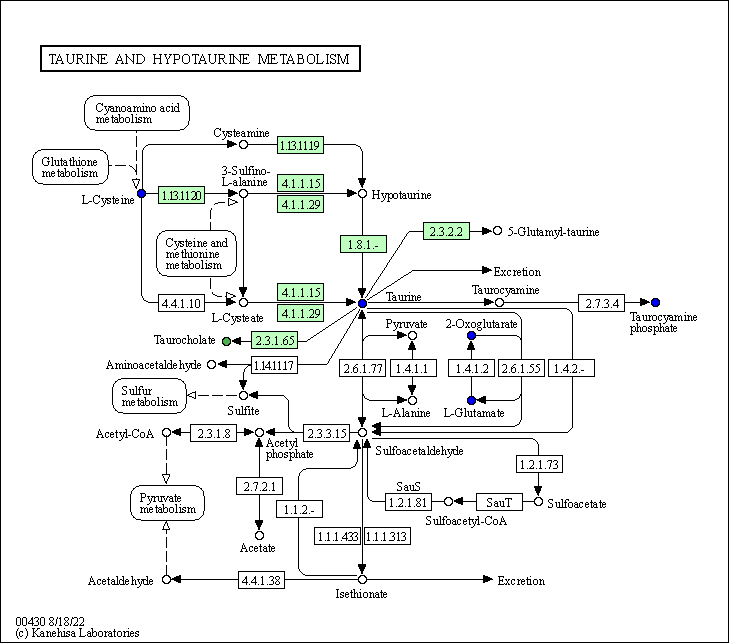

Supplement: Supplementary file 3 [file SupplementaryFile3.zip › 20241027-Supplementary files 4-Figure4-pathway-36σ╝áσ¢╛/ko00430.png]

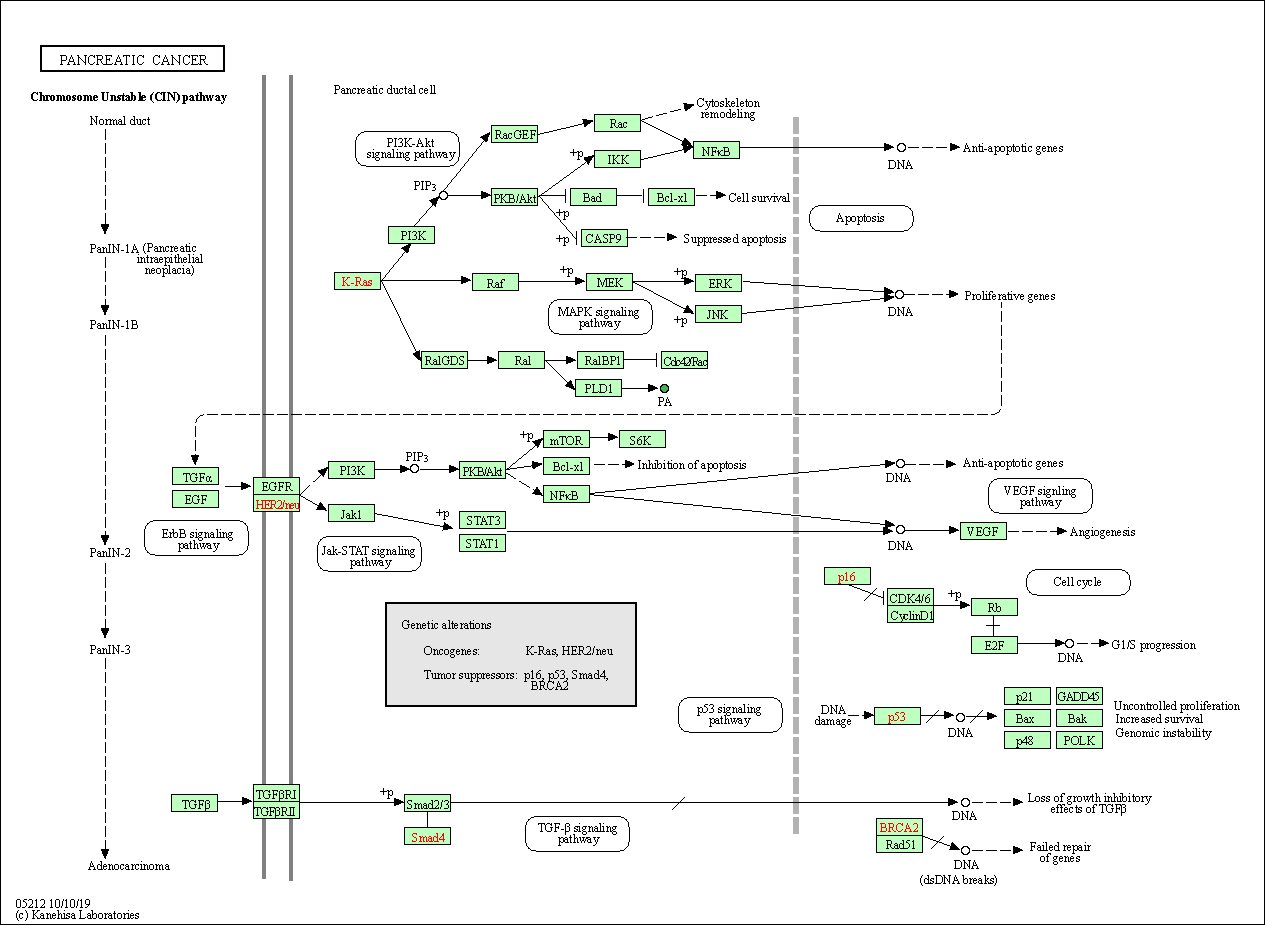

Supplement: Supplementary file 3 [file SupplementaryFile3.zip › 20241027-Supplementary files 4-Figure4-pathway-36σ╝áσ¢╛/ko05212.png]

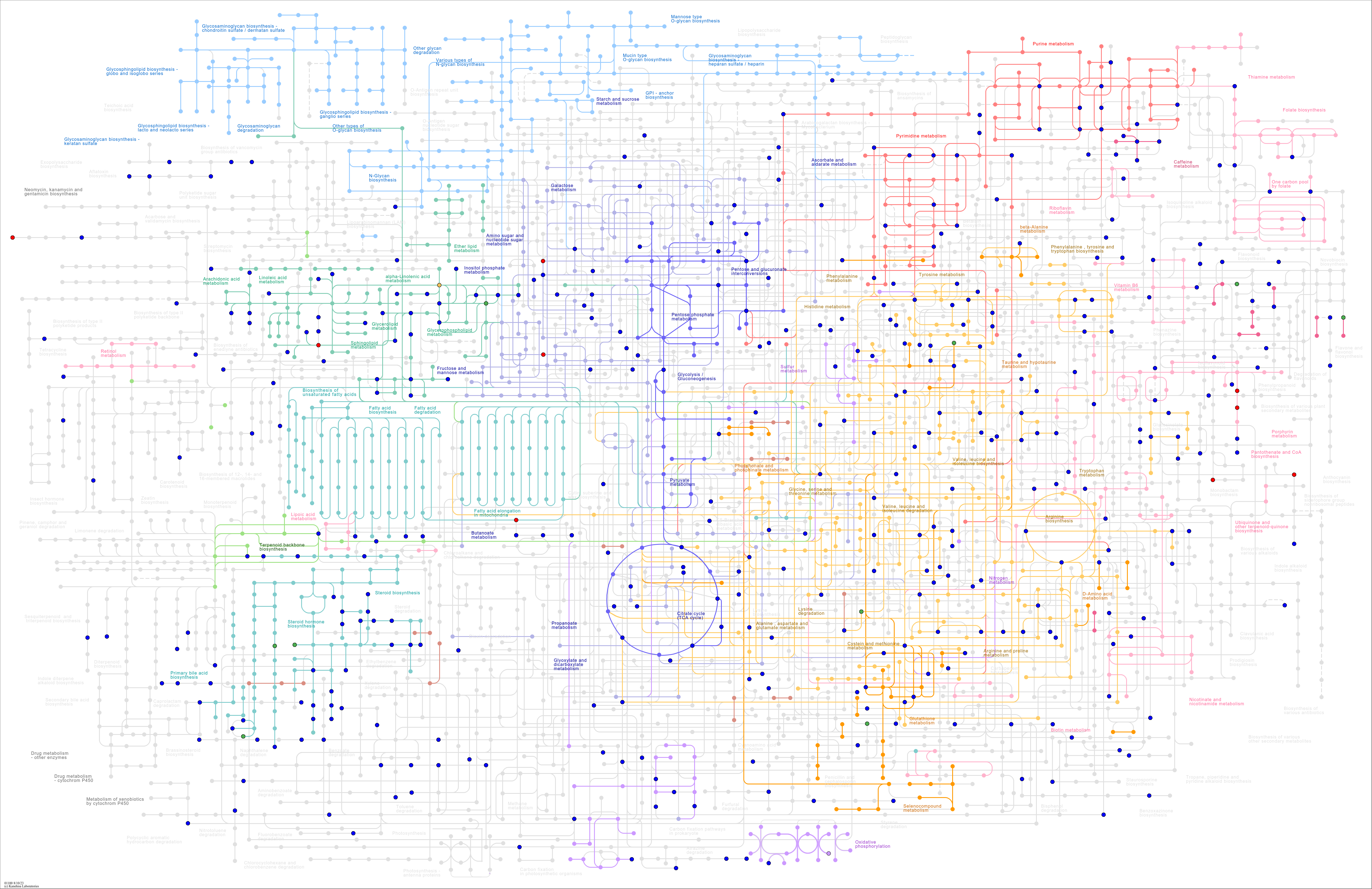

Supplement: Supplementary file 3 [file SupplementaryFile3.zip › 20241027-Supplementary files 4-Figure4-pathway-36σ╝áσ¢╛/ko01100.png]

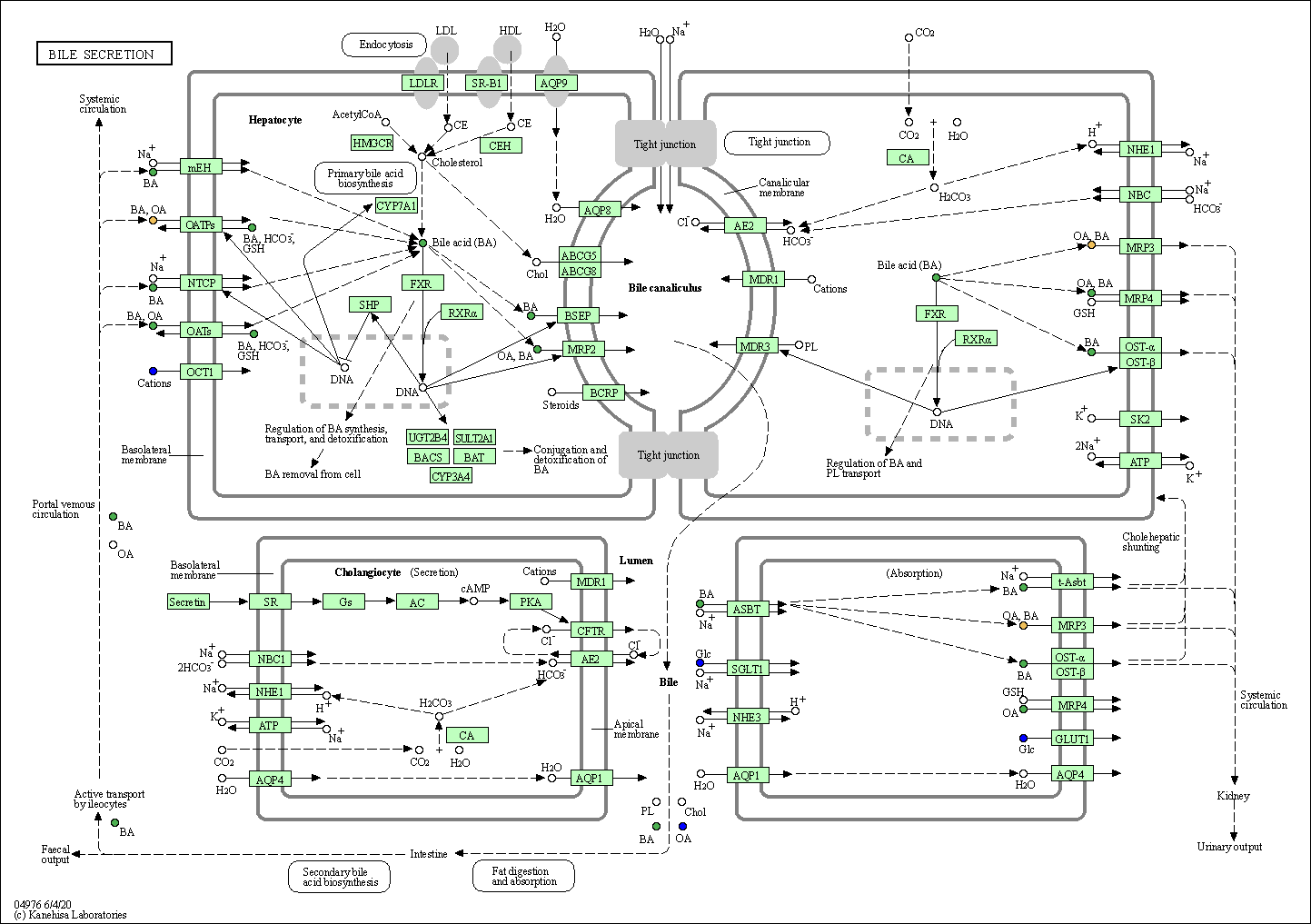

Supplement: Supplementary file 3 [file SupplementaryFile3.zip › 20241027-Supplementary files 4-Figure4-pathway-36σ╝áσ¢╛/ko04976.png]

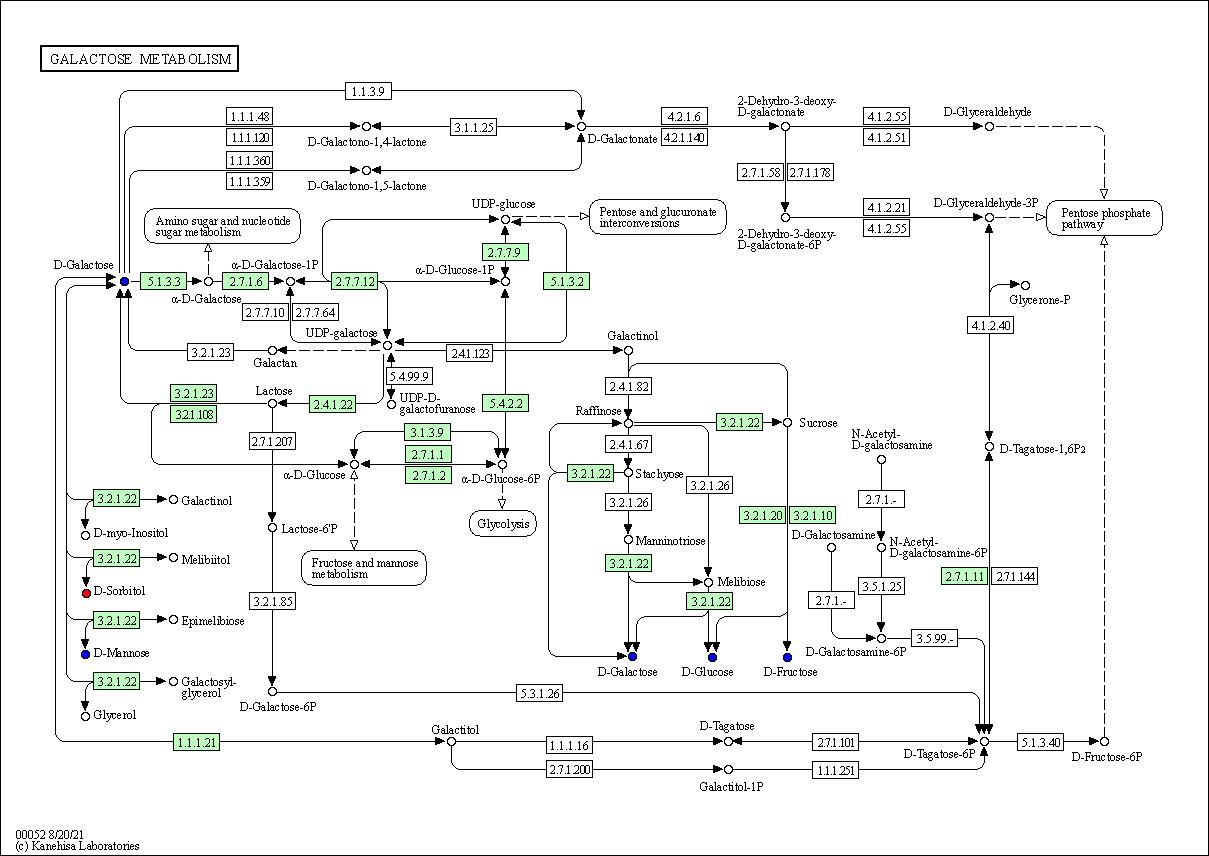

Supplement: Supplementary file 3 [file SupplementaryFile3.zip › 20241027-Supplementary files 4-Figure4-pathway-36σ╝áσ¢╛/ko00052.png]

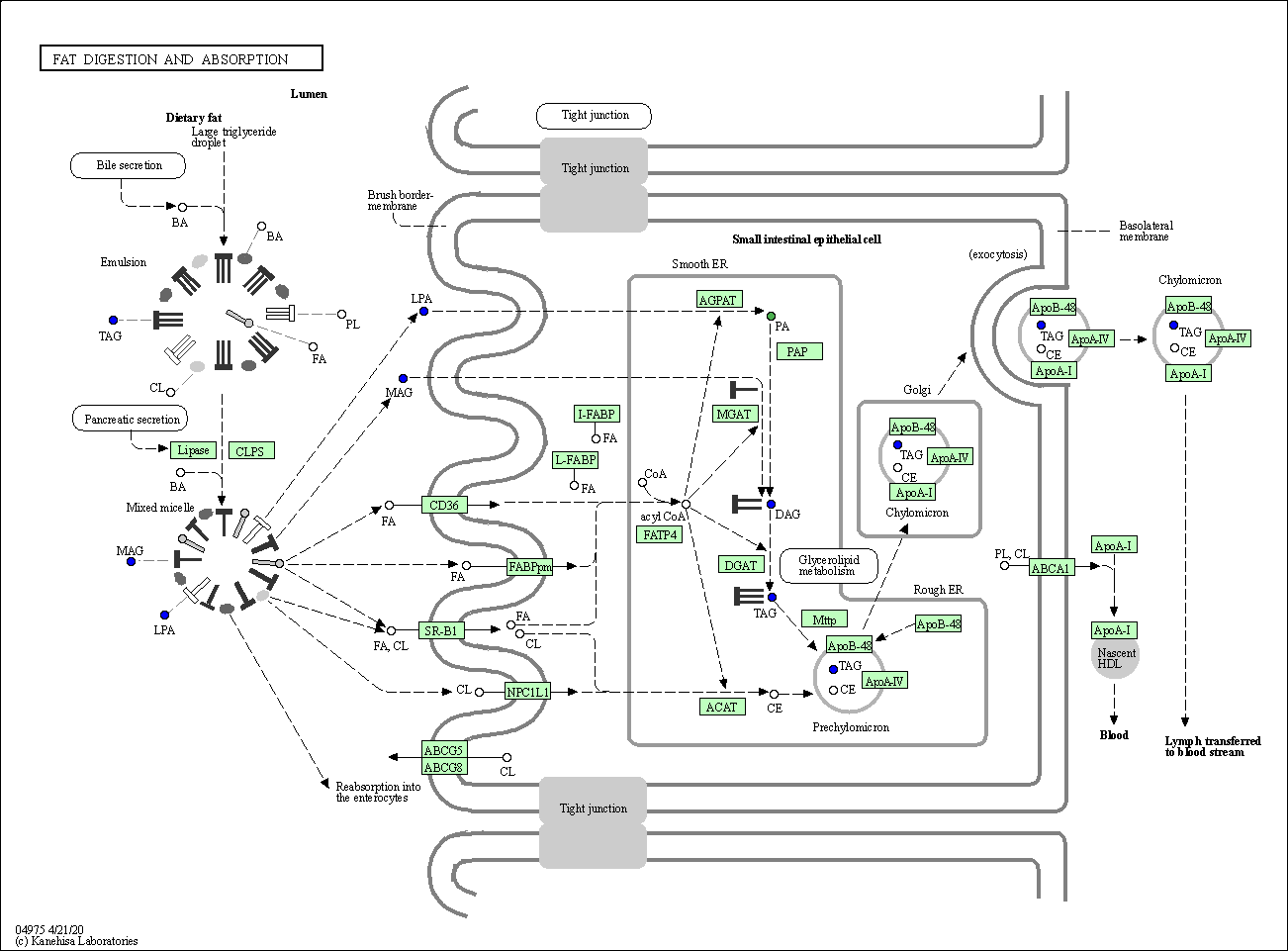

Supplement: Supplementary file 3 [file SupplementaryFile3.zip › 20241027-Supplementary files 4-Figure4-pathway-36σ╝áσ¢╛/ko04975.png]

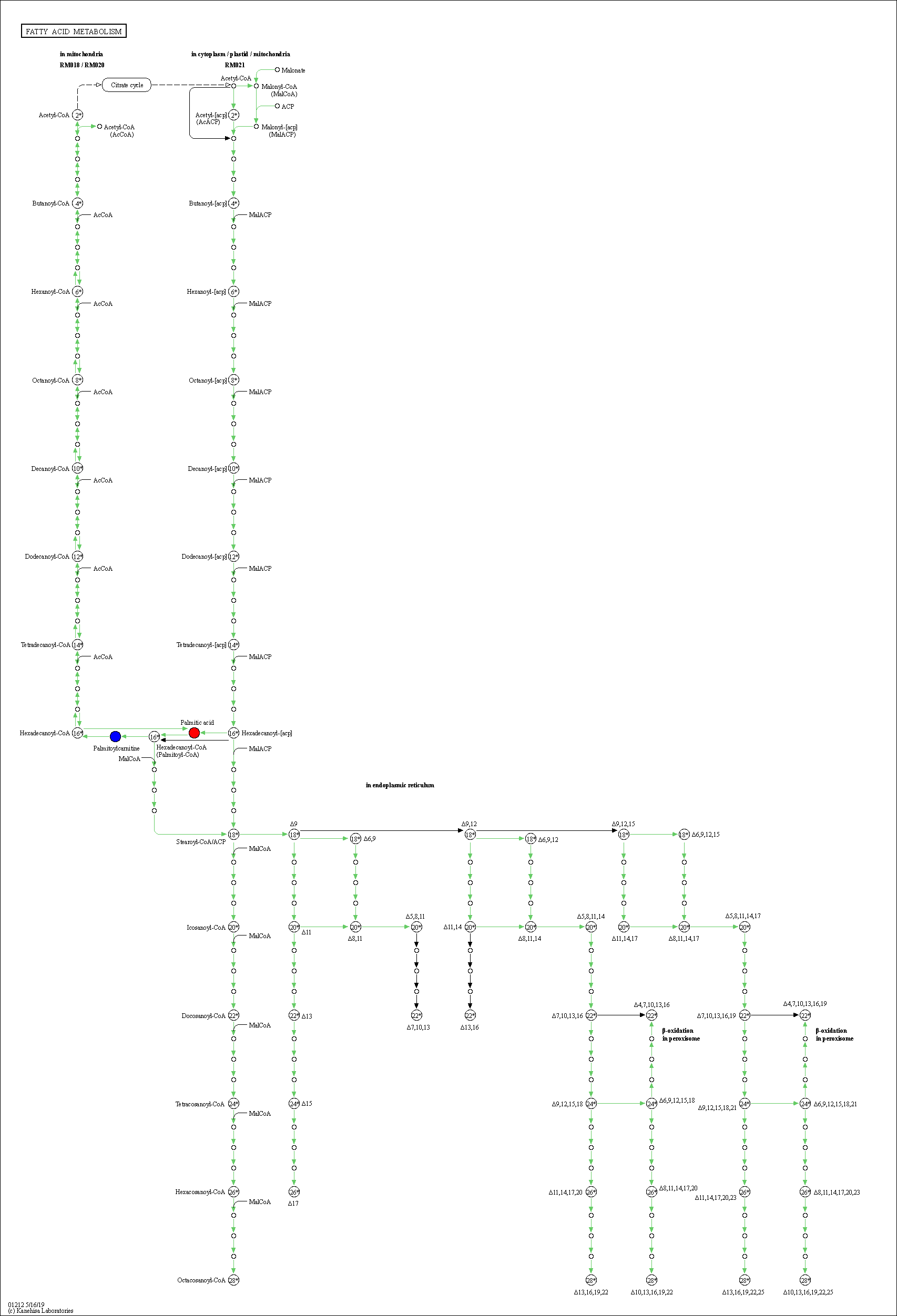

Supplement: Supplementary file 3 [file SupplementaryFile3.zip › 20241027-Supplementary files 4-Figure4-pathway-36σ╝áσ¢╛/ko01212.png]

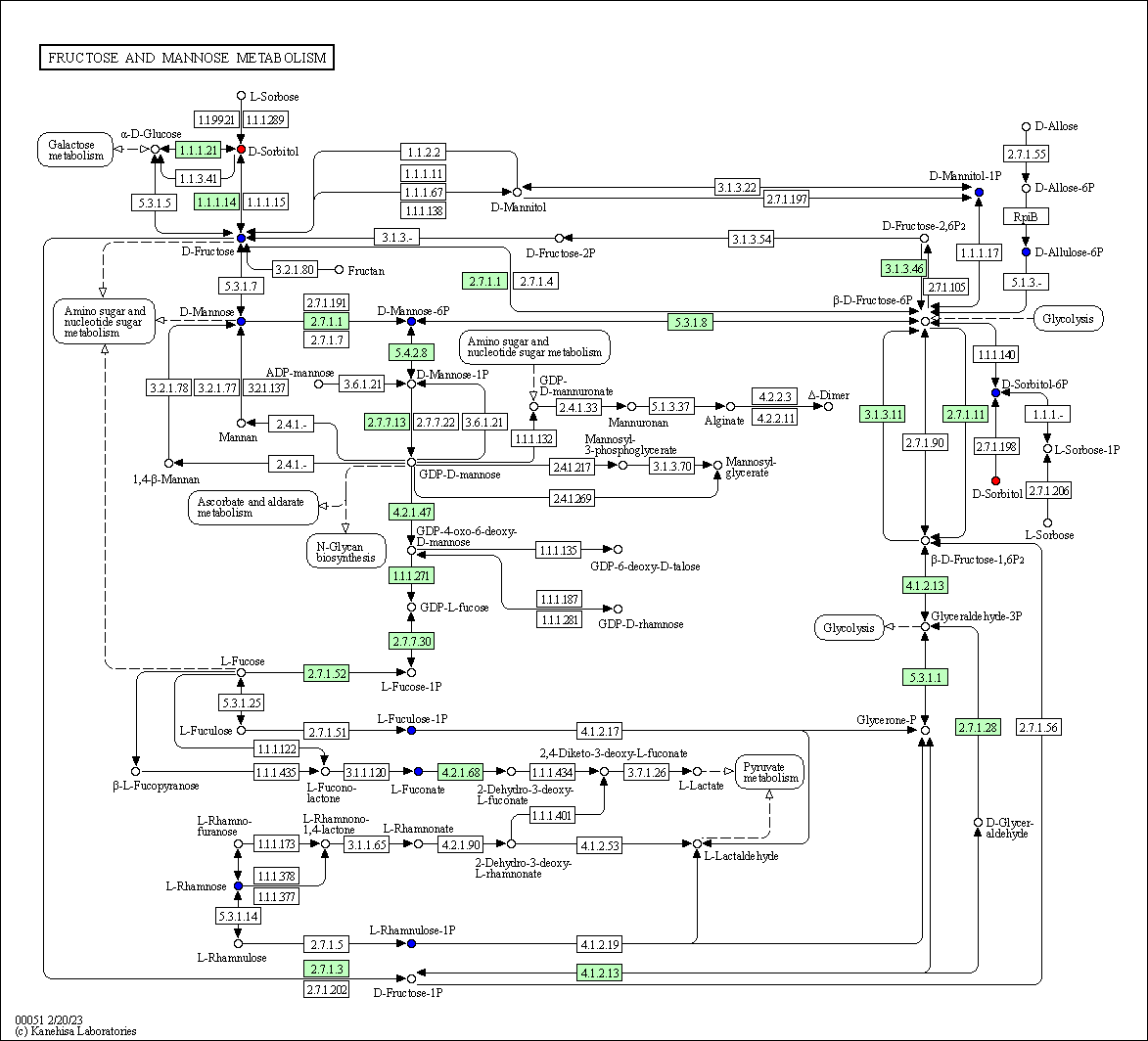

Supplement: Supplementary file 3 [file SupplementaryFile3.zip › 20241027-Supplementary files 4-Figure4-pathway-36σ╝áσ¢╛/ko00051.png]

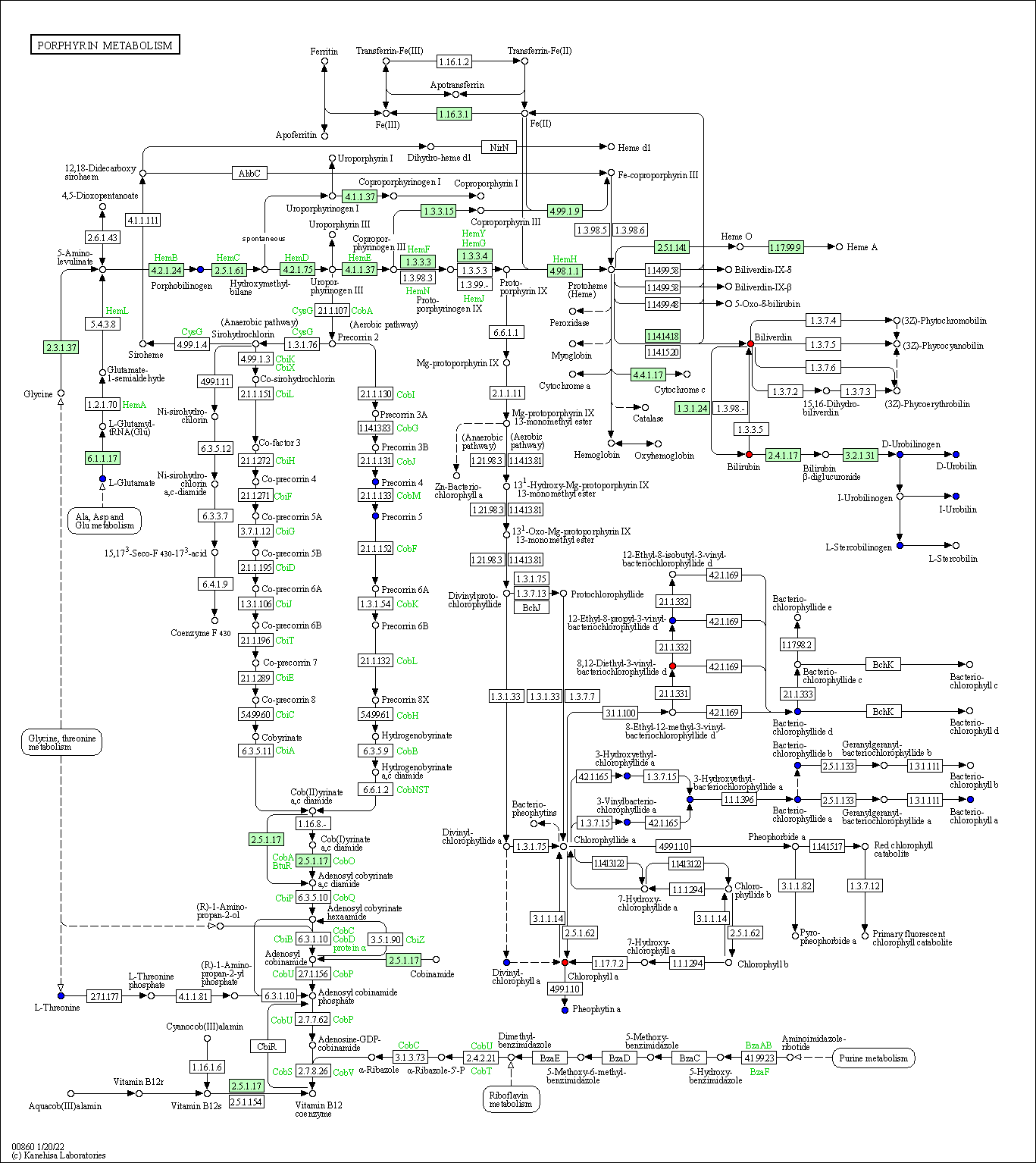

Supplement: Supplementary file 3 [file SupplementaryFile3.zip › 20241027-Supplementary files 4-Figure4-pathway-36σ╝áσ¢╛/ko00860.png]

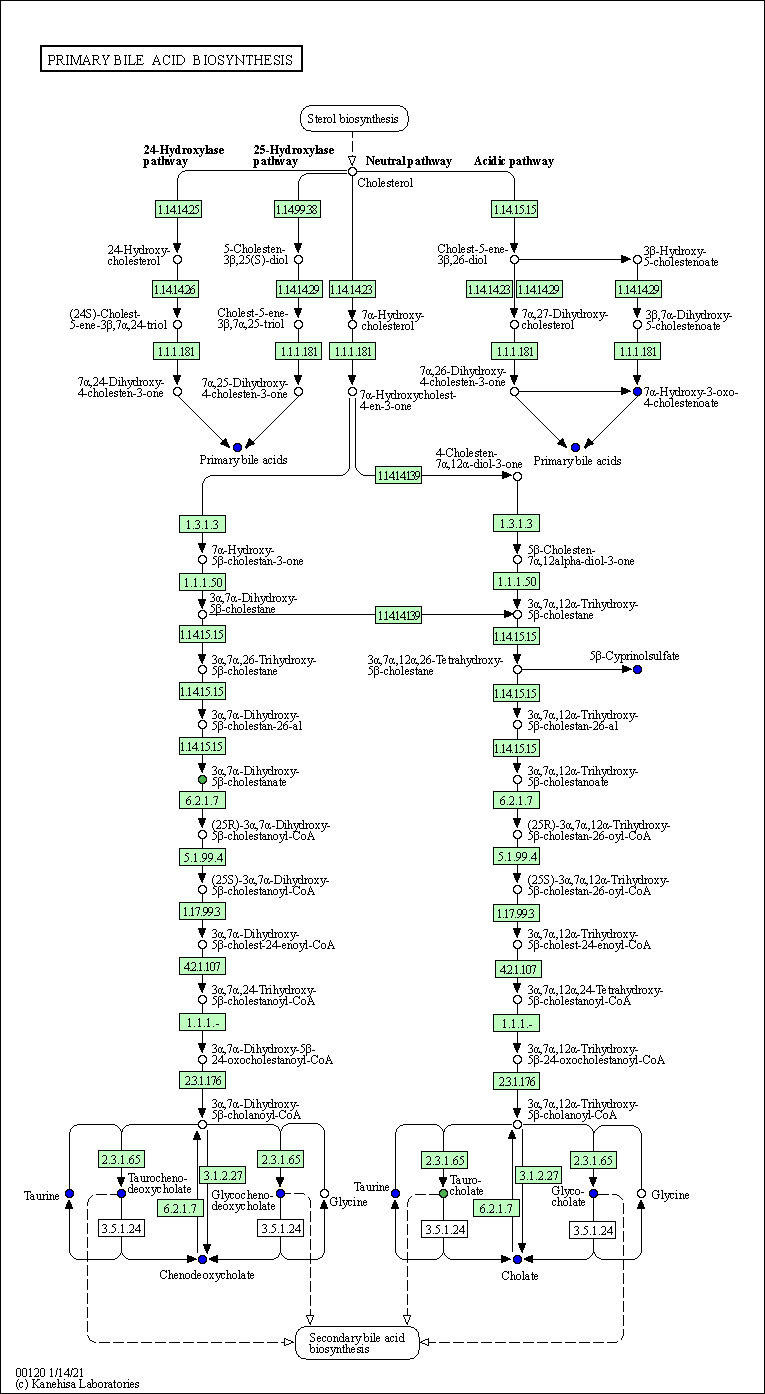

Supplement: Supplementary file 3 [file SupplementaryFile3.zip › 20241027-Supplementary files 4-Figure4-pathway-36σ╝áσ¢╛/ko00120.png]

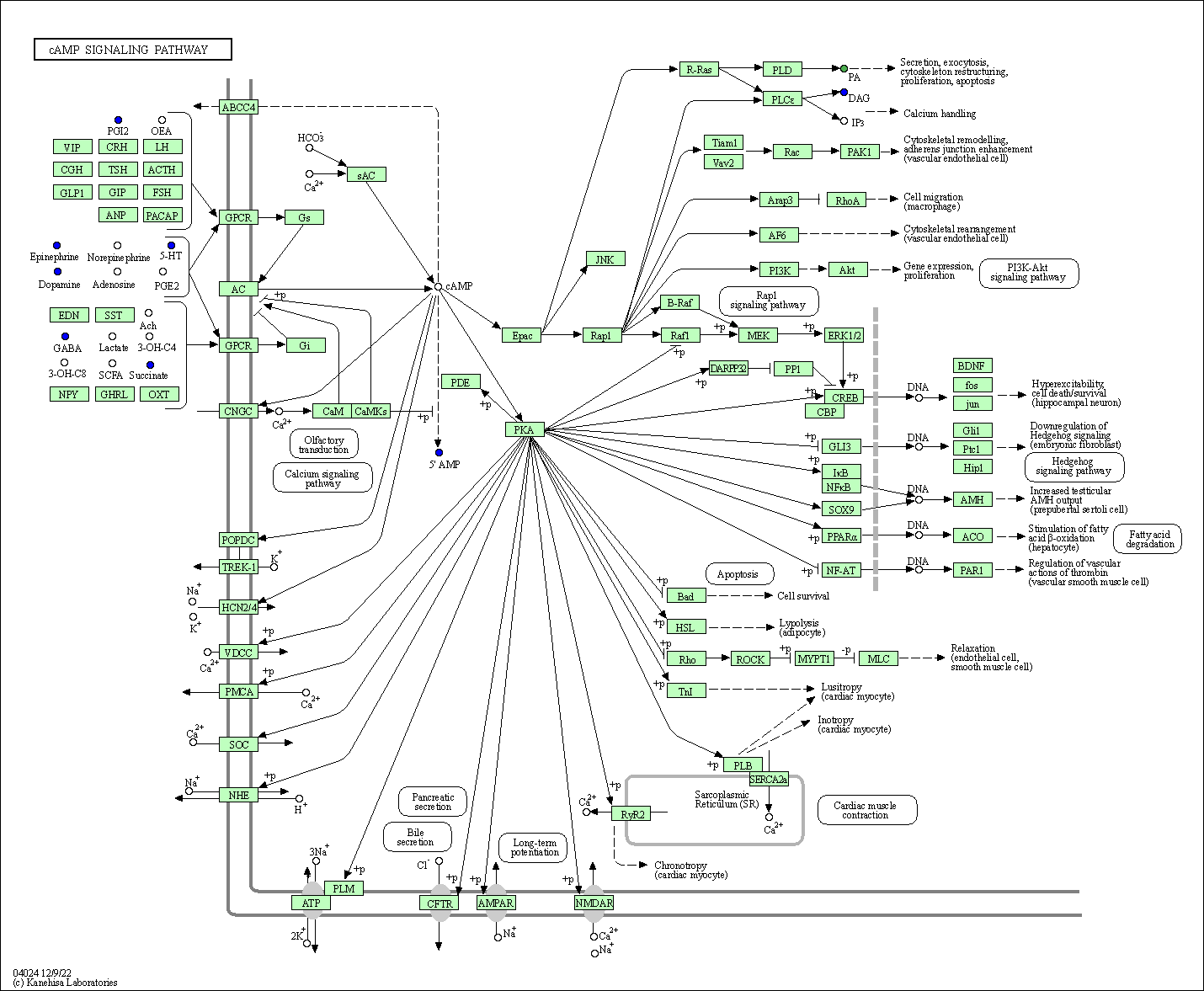

Supplement: Supplementary file 3 [file SupplementaryFile3.zip › 20241027-Supplementary files 4-Figure4-pathway-36σ╝áσ¢╛/ko04024.png]

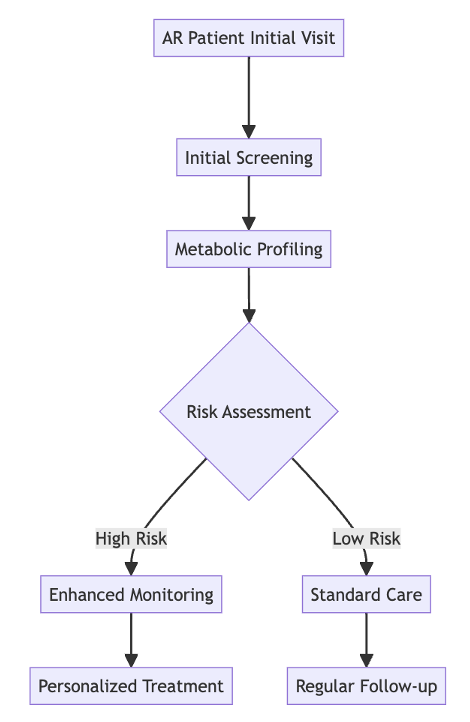

Supplement: Supplementary file 4 [file SupplementaryFile4.jpeg]
